# The TLR7/IRF-5 axis sensitizes memory CD4^+^ T cells to Fas-mediated apoptosis during HIV-1 infection

**DOI:** 10.1172/jci.insight.167329

**Published:** 2023-07-10

**Authors:** Liseth Carmona-Pérez, Xavier Dagenais-Lussier, Linh T. Mai, Tanja Stögerer, Sharada Swaminathan, Stéphane Isnard, Matthew R. Rice, Betsy J. Barnes, Jean-Pierre Routy, Julien van Grevenynghe, Simona Stäger

**Affiliations:** 1 Institut National de la Recherche Scientifique, Centre Armand-Frappier Santé Biotechnologie, and Infectiopôle-INRS, Laval, Quebec, Canada.; 2 Division of Hematology and Chronic Viral Illness Service, McGill University Health Centre, Montreal, Quebec, Canada.; 3 Center for Autoimmune Musculoskeletal and Hematopoietic Diseases, Feinstein Institutes for Medical Research, Manhasset, New York, USA.

**Keywords:** AIDS/HIV, Immunology, Apoptosis, T cells

## Abstract

HIV-1 infection is characterized by inflammation and a progressive decline in CD4^+^ T cell count. Despite treatment with antiretroviral therapy (ART), the majority of people living with HIV (PLWH) maintain residual levels of inflammation, a low degree of immune activation, and higher sensitivity to cell death in their memory CD4^+^ T cell compartment. To date, the mechanisms responsible for this high sensitivity remain elusive. We have identified the transcription factor IRF-5 to be involved in impairing the maintenance of murine CD4^+^ T cells during chronic infection. Here, we investigate whether IRF-5 also contributes to memory CD4^+^ T cell loss during HIV-1 infection. We show that TLR7 and IRF-5 were upregulated in memory CD4^+^ T cells from PLWH, when compared with naturally protected elite controllers and HIV^free^ participants. TLR7 was upstream of IRF-5, promoting Caspase 8 expression in CD4^+^ T cells from ART HIV-1^+^ but not from HIV^free^ donors. Interestingly, the TLR7/IRF-5 axis acted synergistically with the Fas/FasL pathway, suggesting that TLR7 and IRF-5 expression in ART HIV-1^+^ memory CD4^+^ T cells represents an imprint that predisposes cells to Fas-mediated apoptosis. This predisposition could be blocked using IRF-5 inhibitory peptides, suggesting IRF-5 blockade as a possible therapy to prevent memory CD4^+^ T cell loss in PLWH.

## Introduction

Infections with HIV-1 remain a major public health issue. Unlike a number of other viruses, the human body cannot clear HIV, even with antiretroviral therapy (ART), making it a life-long infection (1–3).

One of the hallmarks of HIV infection is the progressive loss of CD4^+^ T cells, which affects both infected and bystander cells. CD4^+^ T cell death occurs via several mechanisms, including direct viral cytopathogenicity, killing by CD8^+^ cytotoxic T lymphocytes, and pyroptosis in infected CD4^+^ T cells or apoptosis in uninfected bystander cells, with the latter being largely Fas/FasL mediated (4–8). Maintenance of a critical count of CD4^+^ T cells is essential to prevent secondary opportunistic infections. ART efficiently controls viral replication, and the majority of people living with HIV (PLWH) are able to restore CD4^+^ T cell count with therapy. Nevertheless, it is still unclear why CD4^+^ T cell homeostasis is not fully normalized in PLWH on ART. More importantly, memory CD4^+^ T cells in PLWH are more prone to Fas-mediated apoptosis (9, 10). The mechanisms leading to this heightened sensitivity are only partially known and involve the proapoptotic activity of the transcription factor Foxo3a (9). Furthermore, residual levels of immune activation and inflammation persist in infected individuals on ART (11, 12).

We have recently identified the transcription factor IFN regulatory factor 5 (IRF-5) as a detrimental player in the survival of CD4^+^ T cells during chronic visceral leishmaniasis (13). IRF-5 is a member of the IFN regulatory factor family, a group of transcription factors with diverse roles, including transcriptional activation of IFNs, and modulation of cell growth, differentiation, apoptosis, and immune system activity (14, 15). In recent years, IRF-5 has gained much attention for its role in regulating inflammatory responses in immunological diseases. IRF-5 is constitutively expressed in antigen-presenting cells, where it is involved in the activation of inflammatory cytokines and type I IFN (IFN-I) production (15–18) and has been identified as a marker for M1 macrophage polarization (19). Moreover, IRF-5 acts as a tumor suppressor (20) and is critical for the induction of apoptosis in response to DNA damage in tumor cells (21). IRF-5 also mediates apoptosis by modulation of proapoptotic genes including DR5, Fas, Caspase 8, DAP kinase 2, Bak, and Bax. (22). In B cells, IRF-5 induces activation, proliferation, and plasmablast differentiation (23) and is required for antibody production and class switching (24). However, while IRF-5 appears to be required for the optimal assembly of the TCR-initiated signaling complex (25), its role in T cells is not yet completely understood.

We have shown that IRF-5 was increasingly expressed during chronic *Leishmania donovani* infection in mice and could be activated via TLR7 expressed in T cells. Interestingly, IFN-γ^+^ CD4^+^ T cells were capable of sensing tissue damage–derived apoptotic cell material via TLR7; activation via TLR7 by apoptotic cell material induced DR5 (or TRAILR2) expression and ultimately led to cell death (13). These findings suggest that sensing of damage-associated molecular patterns (DAMPs) released following tissue damage in a chronic inflammatory environment and the concomitant TLR7 upregulation by CD4 T cells can promote cell death. Since HIV-1 infection is characterized by a strong inflammatory environment, tissue disruption, and upregulation of TLR7 in CD4^+^ T cells (26–29), we wondered whether the TLR7/IRF-5 pathway was involved in impairing memory CD4^+^ T cell homeostasis during HIV-1 infection.

Here, we show that IRF-5 displayed higher levels of expression and activation and that TLR7 was upregulated in CD4^+^ T cells from PLWH when compared with elite controllers (EC) and HIV^free^ participants. Moreover, triggering of TLR7 promoted IRF-5 expression, which in turn induced Caspase 8 expression in CD4^+^ T cells from ART HIV-1^+^ but not from HIV^free^ individuals, sensitizing CD4^+^ T cells to Fas-mediated apoptosis. This cell death pathway could be blocked with IRF-5 blocking peptides. Hence, we propose that heightened expression of TLR7 and IRF-5 in memory CD4^+^ T cells from PLWH on ART represents an imprint that affects memory CD4^+^ T cell homeostasis.

## Results

### IRF-5 is expressed and active in CD4^+^ T cells from primary HIV-infected donors.

We have previously reported that activation of IRF-5 in murine IFN-γ^+^CD4^+^ T cells leads to cell death during chronic visceral leishmaniasis (13). Interestingly, IRF-5 was induced following TLR7 triggering by apoptotic cell material derived from inflammatory tissue disruption, suggesting that DAMP sensing by CD4^+^ T cells may not be microbe specific. Hence, we wondered whether IRF-5 is also expressed and promotes cell death in human CD4^+^ T cells in the context of HIV infection. We chose to analyze the function of IRF-5 in CD4^+^ T cells from PLWH, since the infection is associated with a strong inflammatory environment and with a loss of CD4^+^ T cells before ART initiation (primary HIV-1–infected [PHI] phase), and because TLR7 is expressed on CD4^+^ T cells during HIV-1 infection (28). PHI participants were between 27 and 56 years old and displayed viral loads ranging from 3.08 ***×*** 10^3^ to 513.54 ***×*** 10^3^ copies/mL, and their viral acquisition was estimated to have occurred less than 4 months prior to sample collection. Age-matched uninfected participants were selected as a negative control. The demographic and clinical characteristics of each group are summarized in Table 1.

Before we proceeded to analyze IRF-5 expression in CD4^+^ T cells from PHI participants, we tested the anti–human IRF-5 antibody from R&D Systems for its specificity, which has been a problem for many commercial antibodies recognizing IRF-5 (30). To this end, we activated IRF-5–sufficient and –deficient THP-1 cells with imiquimod (IMQ) or PMA + IMQ. More than half of the unstimulated THP-1 cells constitutively expressed IRF-5; this percentage increased upon stimulation with IMQ, and nearly all the cells expressed IRF-5 after incubation with PMA + IMQ (Supplemental Figure 1A; supplemental material available online with this article; https://doi.org/10.1172/jci.insight.167329DS1). In contrast, we did not detect IRF-5 expression in *IRF5^–/–^* THP-1 cells even after stimulation with IMQ or PMA + IMQ (Supplemental Figure 1A), suggesting that this anti–human IRF-5 antibody is specific. We also determined IRF-5 nuclear localization by ImageStreamX (Supplemental Figure 1B).

**Figure 1:**
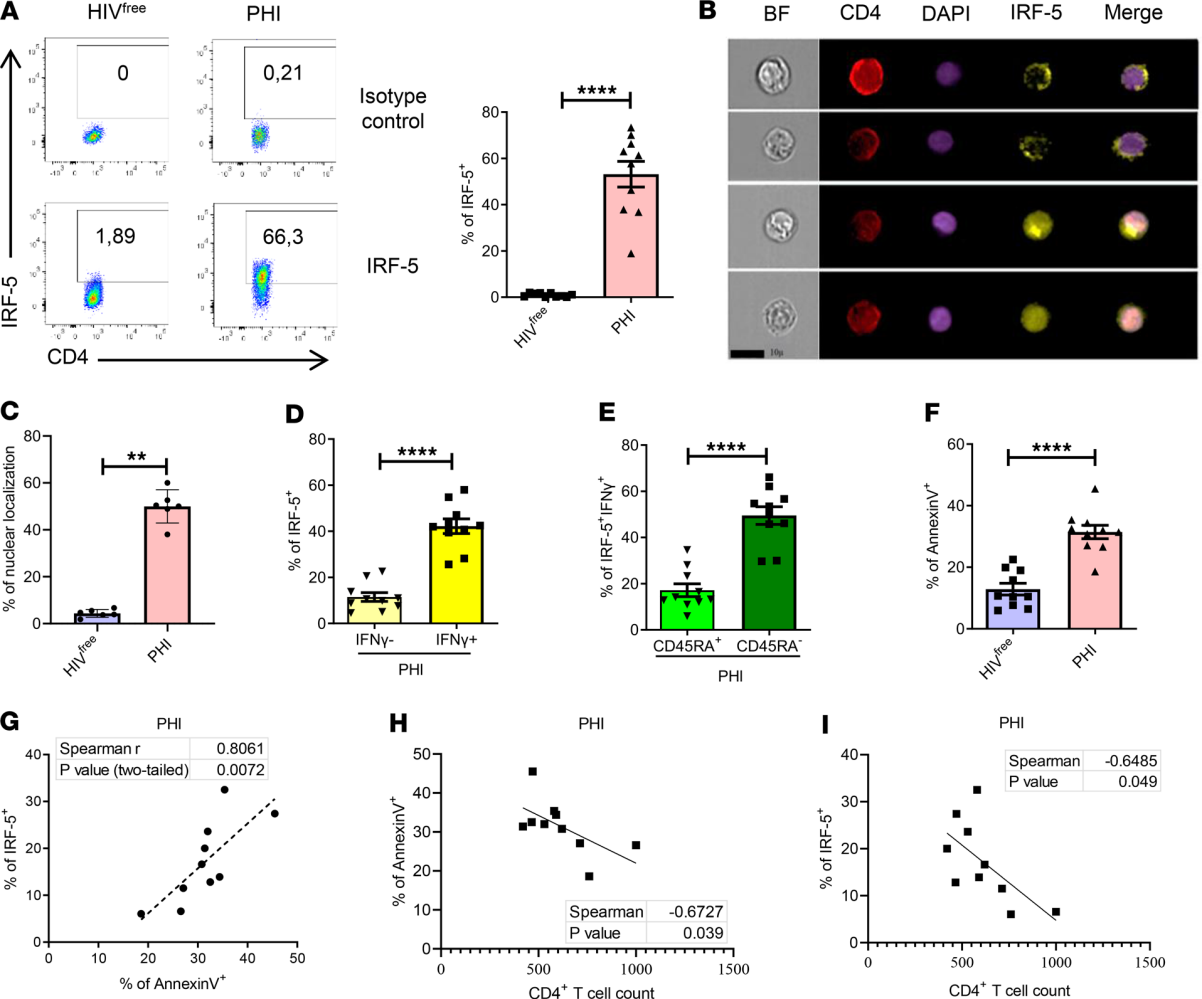
IRF5 is expressed in CD4^+^ T cells from PHI. PBMCs from PHI and HIV^free^ individuals were analyzed by flow cytometry. (**A**) Representative FACS plots and percentage of IRF-5^+^ CD4^+^ T cells. (**B**) Representative ImageStreamX images of CD4^+^ T cells expressing IRF5 in the cytoplasm (first 2 lanes) and in the nucleus (third and fourth lane). (**C**–**F**) Graphs show (**C**) the frequency of CD4^+^ T cells whose IRF-5 expression colocalizes with the nucleus; (**D**) the percentage of IFN-γ^+^ and IFN-γ^–^IRF-5^+^ CD4^+^ T cells; (**E**) the frequency of CD45RA^+^ and CD45RA^–^IFN-γ^+^ CD4^+^ T cells expressing IRF-5 in PHI individuals; and (**F**) the percentage of annexin V^+^CD4^+^ T cells from PHI and HIV^free^ individuals. Data are presented as the mean ± SD. The Mann-Whitney *U* test was used for significance. **P < 0.01, ****P < 0.0001, *n* =10. Graphs represent correlations between (**G**) annexin V and IRF-5 expression in CD4^+^ T cells, (**H**) annexin V expression and CD4^+^ T cell count, and (**I**) IRF-5 expression and CD4^+^ T cell count in PHI donors. The Spearman’s *r* test was used for significance, *P < 0.05, **P < 0.01, *n* =10.

We found a higher frequency of IRF-5–expressing CD4^+^ T cells in PHI participants when compared with CD4^+^ T cells from HIV^free^ control participants (Figure 1A; gating strategy is shown in Supplemental Figure 1C). Moreover, this transcription factor colocalized with the nucleus in about 40%–60% of IRF-5^+^ CD4^+^ T cells from HIV-1^+^ donors compared with 1.8%–6.7% in CD4^+^ T cells from HIV^free^ control participants (Figure 1, B and C). As in our previous observations (13), the majority of cells expressing IRF-5 also secreted IFN-γ (Figure 1D) and were mainly effector and memory cells (Figure 1E and Supplemental Figure 2A). As expected, CD4^+^ T cells from PLWH also showed higher frequencies of annexin V^+^ cells, a common marker of cell death and apoptosis, compared with cells from HIV^free^ control participants (Figure 1F). Of note, IRF-5 expression positively correlated with annexin V expression (Figure 1G) and annexin V expression inversely correlated with CD4^+^ T cell count in PLWH (Figure 1H). Moreover, IRF-5 expression also inversely correlated with CD4^+^ T cell count (Figure 1I), suggesting that this transcription factor could contribute to cell death.

In mice, IRF-5 has been shown to promote cell death by inducing the expression of death receptor 5 (DR5) (13). However, we did not observe any difference in DR5 expression between CD4^+^ T cells from PHI and HIV^free^ donors (Supplemental Figure 2B), even though DR5^+^ cells were mostly IRF-5^+^ (Supplemental Figure 2C) and nearly all IFN-γ^+^ cells were also DR5^+^ (Supplemental Figure 2D). Moreover, DR5 expression did not correlate with annexin V expression (Supplemental Figure 2E), suggesting that, contrary to what was observed in *L*. *donovani*–infected mice (13), DR5 is most likely not involved in CD4^+^ T cell death in PHI individuals.

### ART HIV-1^+^ individuals show higher frequencies of IRF-5–expressing CD4^+^ T cells.

PLWH treated with ART still show low grade inflammation (11, 12), and their memory CD4^+^ T cells are prone to Fas-mediated apoptosis, despite years of aviremia in the blood compartment (9, 10). Thus, we next wanted to know whether IRF-5 is also expressed in memory CD4^+^ T cells from PLWH undergoing ART and is involved in sensitizing memory CD4^+^ T cells to apoptosis.

PLWH on ART who participated in this study displayed both viral suppression (viral loads < 40 copies/mL) and CD4^+^ T cell recovery (> 400 cells/μL in blood after treatment) for at least 5 years. EC participants had undetectable viral loads (<40 copies/mL) without treatment for more than 5 years, with blood CD4^+^ T cell counts over 400 cells/μL. The demographic and clinical characteristics of each study group are summarized in Table 1.

**Figure 2:**
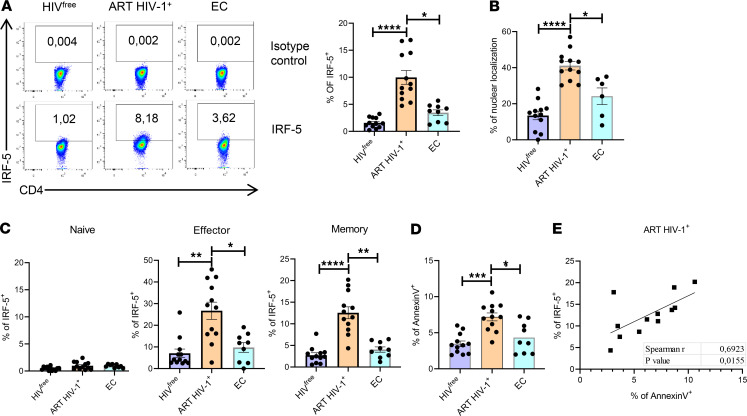
IRF5 is expressed in memory and effector CD4^+^ T cells from individuals on ART. PBMCs from ART HIV-1^+^, EC, and HIV^free^ donors were analyzed by flow cytometry. (**A**) Representative FACS plots and percentage of IRF-5^+^ CD4^+^ T cells. (**B**–**D**) Graphs show (**B**) the percentage of CD4^+^ T cells expressing IRF-5 in the nucleus; (**C**) the percentage of IRF-5 expression in naive, effector, and memory CD4^+^ T cell; and (**D**) the percentage of memory CD4^+^ T cells positive for annexin V in PBMCs isolated from ART HIV-1^+^, EC, and HIV^free^ individuals. Data are presented as the mean ± SD. The Kruskal-Wallis followed by the Dunn’s multiple-comparison tests were used for significance. *P < 0.05, **P < 0.01, ***P < 0.001, ****P < 0.0001, *n* = 12 (ART HIV-1^+^ and HIV^free^), *n* = 9 (EC). (**E**) Graph represents correlation between annexin V and IRF-5 expression in memory CD4^+^ T cells from patients with HIV-1^+^ on ART. The Spearman’s *r* test was used for significance. *n* =12.

We found significantly higher frequencies of IRF-5–expressing CD4^+^ T cells in ART HIV-1^+^ individuals (Figure 2A) compared with controls. Moreover, IRF-5 colocalized with the nucleus in nearly the half of the IRF-5^+^ cells (Figure 2B; gating strategy is shown in Supplemental Figure 3). Since HIV-1^+^ ECs can control plasma viral load while maintaining CD4^+^ T cell count in the absence of ART, we were interested in determining the percentage of IRF-5–expressing CD4^+^ T cells isolated from EC donors. In contrast to ART HIV-1^+^ individuals, EC presented with lower frequencies of IRF-5^+^CD4^+^ T cells (Figure 2A), with IRF-5 being active in about 20%–25% of the IRF-5^+^ cells (Figure 2B). Independently of expression levels, IRF-5 was mostly expressed in effector and memory CD4^+^ T cells in all 3 study groups (Figure 2C). Since IRF-5 is expressed in memory CD4^+^ T cells and these cells are more prone to apoptosis in ART HIV-1^+^ individuals, we decided to focus on memory CD4^+^ T cells. Interestingly, memory CD4^+^ T cells from ART HIV-1^+^ but not EC donors expressed significantly higher levels of annexin V (Figure 2D) when compared with the uninfected control group, suggesting that memory CD4^+^ T cell death was more frequent in ART HIV-1^+^ individuals. Expression of annexin V in memory CD4^+^ T cells correlated with IRF-5 expression (Figure 2E), suggesting that this transcription factor may contribute to memory cell death in ART HIV-1^+^ donors. No correlation was found between IRF-5 and annexin V expression in memory cells from ECs (Supplemental Figure 4A).

### TLR7 is upstream of IRF-5 in memory CD4^+^ T cells from ART HIV-1^+^ individuals.

**Figure 3:**
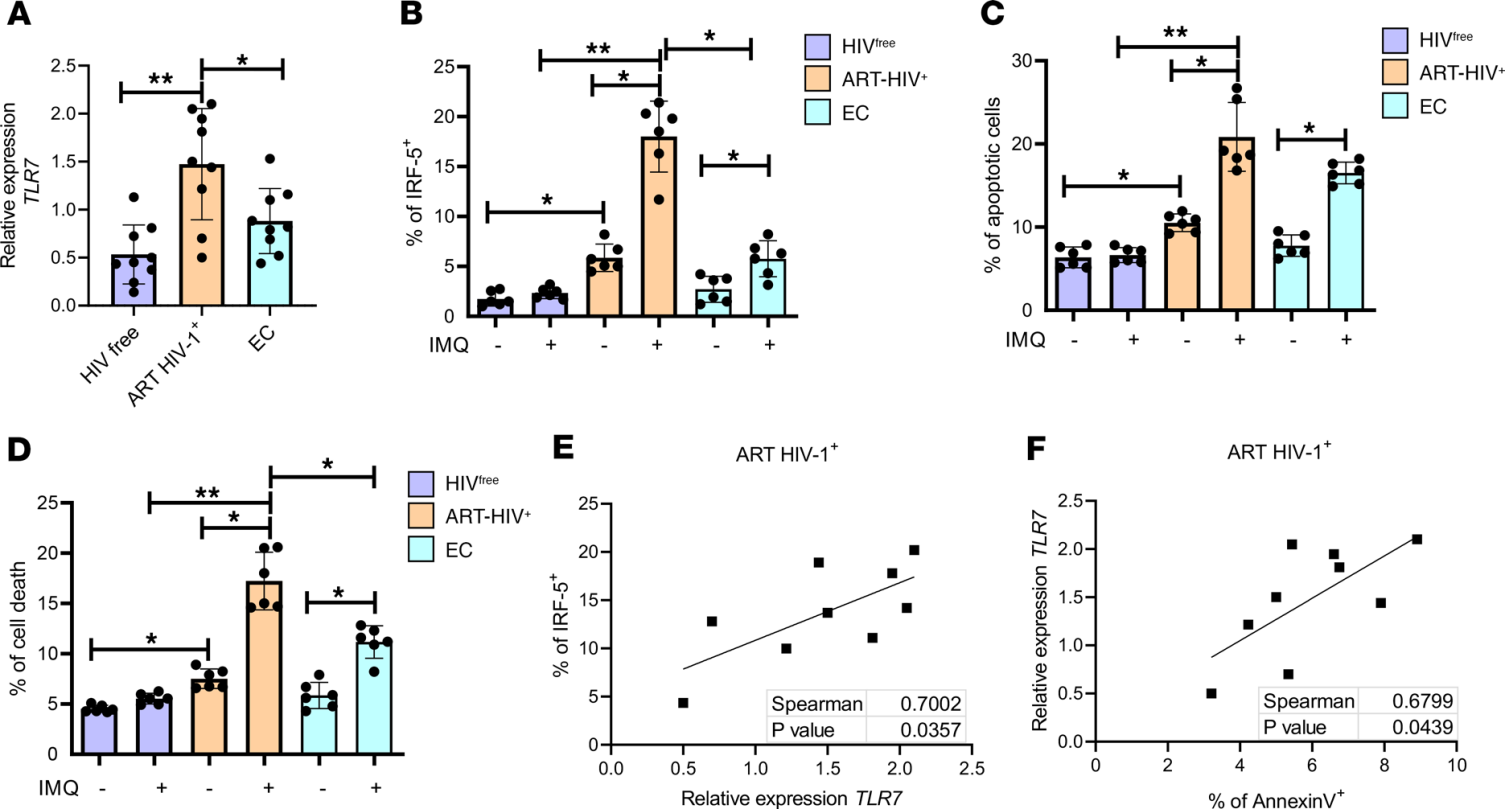
TLR7 is upstream of IRF5. (**A**) Graph shows *TLR7* mRNA levels in CD4^+^ T cells from ART HIV-1^+^, EC, and HIV^free^ individuals analyzed by qPCR. The Kruskal-Wallis followed by the Dunn’s multiple-comparison tests were used for significance. *P < 0.05, **P < 0.01. *n* =9. CD4^+^ T cells purified from ART HIV-1^+^, EC, and HIV^free^ donors were treated in vitro with imiquimod (IMQ) or medium alone for 30 hours. (**B**–**D**) Graphs represent (**B**) the percentage of IRF-5^+^, (**C**) the percentage of apoptotic cells, and (**D**) the percentage dead memory CD4^+^ T cells. Data are presented as the mean ± SD. The Friedman’s followed by the Dunn’s multiple-comparison tests were used to determine statistical differences. *P < 0.05, **P < 0.01. *n* = 6. (**E** and **F**) Graphs show correlations between (**E**) *TLR7* and IRF-5 expression and (**F**) annexin V and TLR7 expression in ART HIV-1^+^ donors. The Spearman’s *r* test was used for statistical significance. *n* =9.

We next wanted to investigate IRF-5 upstream signaling partners in memory CD4^+^ T cells. We have previously demonstrated that TLR7 was upregulated in murine CD4^+^ T cells during chronic *L. donovani* infection and was upstream of IRF-5 (13). TLR7 is known to be expressed in human CD4^+^ T cells during HIV-1 infection (28), but it is still unknown whether TLR7 expression is maintained in CD4^+^ T cells after ART. Hence, we analyzed *TLR7* mRNA expression in CD4^+^ T cells isolated from ART HIV-1^+^, EC, and HIV^free^ individuals. The highest levels of *TLR7* mRNA expression were detected in CD4^+^ T cells from ART HIV-1^+^ donors, while ECs expressed intermediate *TLR7* levels compared with HIV^free^ donors (Figure 3A). Subsequently, we stimulated CD4^+^ T cells from the different study groups with IMQ, a TLR7 agonist. We found that treatment of the cells with IMQ induced IRF-5 expression in memory CD4^+^ T cells from ART HIV-1^+^ and, to a lesser extent, from EC donors but not in HIV^free^ individuals (Figure 3B). To understand what would trigger TLR7 and activate IRF-5 in vivo, we measured regenerating islet-derived protein 3 α (REG3α) in the serum of participants as a marker for intestinal mucosa damage (31). In agreement with the literature, we found a significant elevation of REG3α in the serum of male and female ART HIV^+^ donors, while this molecule was detected at very low levels in sera from HIV^free^ donors (Supplemental Figure 4B). Moreover, we found a significant correlation between the frequency of IRF-5 expression and the amount of REG3α in the serum of ART HIV^+^ donors (Supplemental Figure 4C), implying that DAMPs released from the damaged intestinal mucosa could trigger TLR7 and induce IRF-5.

In agreement with our previous findings (13), IMQ treatment induced apoptosis (Zombie Aqua^–^annexin V^+^Caspase 3^+^) (Figure 3C) and cell death (Zombie Aqua^+^annexin V^+^) (Figure 3D) in memory CD4^+^ T cells from ART HIV-1^+^ individuals to a significantly higher degree than in memory CD4^+^ T cells from EC, directly correlating with TLR7 expression levels. Of note, IMQ treatment did not affect cell survival of CD4^+^ T cells from HIV^free^ individuals (Figure 3D). Our results suggest that the TLR7/IRF-5 axis could play a role in memory CD4^+^ T cell death in ART HIV-1^+^ donors. This hypothesis was supported by the direct correlation between TLR7 and IRF-5 expression (Figure 3E) and between annexin V and TLR7 expression (Figure 3F). Although TLR7 and IRF5 are also expressed in CD4^+^ T cells from ECs, no correlation between the expression of IRF-5 and TLR7 and between the expression of TLR7 and annexin V was found in these cells (Supplemental Figure 5, A and B).

CD4^+^ T cell stimulation via TCR has been reported to induce IRF-5 in mice (25). We found that the stimulation of cells with αCD3/αCD28 also induced IRF-5 in total (Supplemental Figure 5D) and memory CD4^+^ T cells (Supplemental Figure 5E) from ART HIV-1^+^ and HIV^free^ individuals. However, this did not promote apoptosis (Supplemental Figure 5F) or cell death (Supplemental Figure 5G).

### Caspase 8 is downstream of the TLR7/IRF-5 pathway.

**Figure 4:**
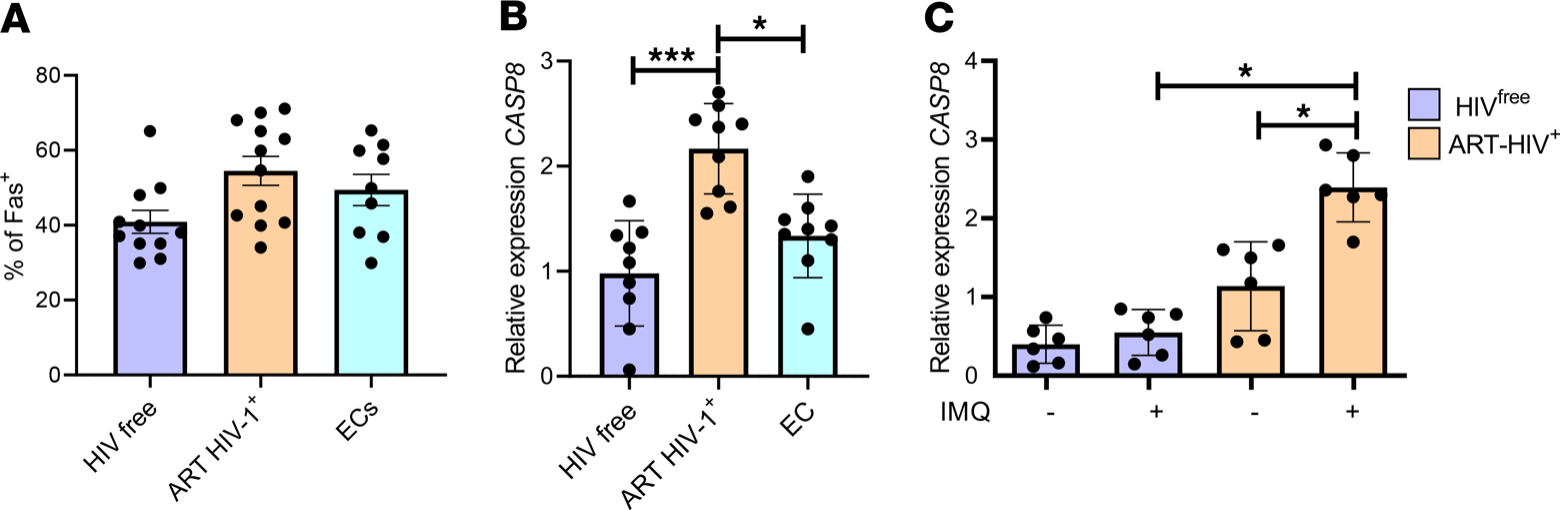
Caspase 8 is downstream of IRF5. CD4^+^ T cells from PBMCs of ART HIV-1^+^, EC, and HIV^free^ individuals were analyzed ex vivo. (**A** and **B**) Graphs show (**A**) the percentage of Fas^+^ memory CD4^+^ T cells determined by flow cytometry and (**B**) RT-PCR analysis of *CASP8* mRNA levels in purified CD4^+^ T cells. Data are presented as the mean ± SD. The Kruskal-Wallis followed by the Dunn’s multiple-comparison tests were used for significance. *P < 0.05, ***P < 0.001. *n* =12 (ART HIV-1^+^ and HIV^free^), *n* =9 (EC). (**C**) Purified CD4^+^ T cells from ART HIV-1^+^ and HIV^free^ donors were treated in vitro with imiquimod (IMQ) or medium alone for 30 hours. Graph show expression of *CASP8* mRNA. Data are presented as the mean ± SD. The Friedman’s followed by the Dunn’s multiple-comparison tests were used for significance. *P < 0.05. *n* =6.

Several molecules involved in cell death have been described to be downstream molecular targets of IRF-5, including Fas, DR5, and Caspase 8, among others (22). Since it was previously reported that memory CD4^+^ T cell death during HIV-1 infection is Fas-mediated (9), and IRF-5 is required for Fas-induced apoptosis (32), we set out to investigate whether Fas was downstream of IRF-5 in human CD4^+^ T cells. In agreement with the literature (9), we found no difference in the expression of Fas between any of the groups studied (Figure 4A). Hence, we investigated whether Caspase 8 expression differed between the various study groups. A significant upregulation of *CASP8* mRNA levels was observed in CD4^+^ T cells from ART HIV-1^+^ donors when compared with EC and HIV^free^ individuals (Figure 4B). Moreover, when we treated CD4^+^ T cells with IMQ, memory CD4^+^ T cells from ART HIV-1^+^ but not HIV^free^ individuals upregulated *CASP8* mRNA expression (Figure 4C). Taken together, our results suggest that the TLR7/IRF-5 axis induces Caspase 8 expression and appears to be active in memory CD4^+^ T cells from ART HIV-1^+^ donors.

### Activation of the TLR7/IRF-5 axis predisposes cells to Fas-mediated apoptosis.

**Figure 5:**
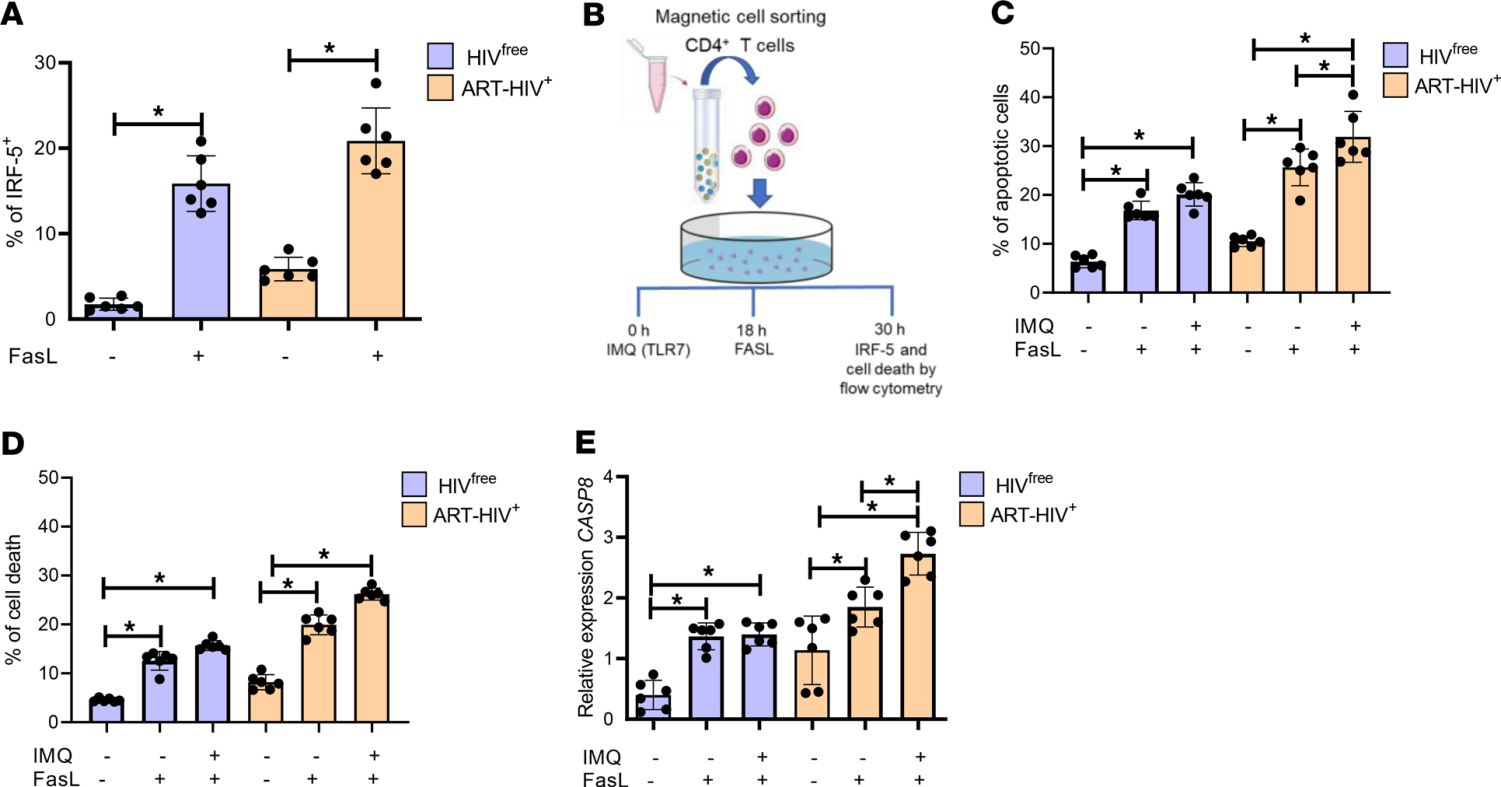
Activation of the TLR7/IRF-5 axis predisposes memory CD4^+^ T cells to Fas-mediated apoptosis. (**A**) Purified CD4^+^ T cells from ART HIV-1^+^ and HIV^free^ donors were incubated in vitro with rFasL or medium alone for 18 hours. Graph shows IRF-5 expression as measured by flow cytometry. Data are presented as the mean ± SD. The Friedman’s followed by the Dunn’s multiple-comparison tests were used for significance. *P < 0.05. *n* =6. (**B**) Scheme of the experimental design. Purified CD4^+^ T cells were treated with IMQ or medium alone; 12 hours later, rFasL or medium was added to the culture, and the cells were incubated for a further 18 hours for a total of 30 hours of incubation at 37°C. (**C**–**E**) Graphs show (**C**) the percentage of apoptotic memory CD4^+^ T cells, (**D**) the percentage of dead memory CD4^+^ T cells, and (**E**) *CASP8* mRNA levels in memory CD4^+^ T cells after 30-hour stimulation. Data are presented as the mean ± SD. The Wilcoxon test was used for significance. *P < 0.05. *n* =6.

IRF-5 has been shown to be required for Fas-mediated cell death (32). Therefore, we investigated whether Fas/FasL interaction could also induce IRF-5. When we treated CD4^+^ T cells with recombinant human Fas ligand (rFasL), we observed higher percentages of IRF-5^+^ memory CD4^+^ T cells in both ART HIV-1^+^ and HIV^free^ individuals (Figure 5A). Since IRF-5 and Caspase 8 are also part of the Fas/FasL signaling pathway (32–34), we hypothesized that activation of the TLR7/IRF-5/Caspase 8 axis would predispose cells to Fas-mediated apoptosis and enhance cell death. To prove our hypothesis, we pretreated cells with or without IMQ for 18 hours, followed by a 12-hour incubation with rFasL, and we finally analyzed the frequency of apoptotic and dead memory CD4^+^ T cells (Figure 5B). As expected, we detected significantly higher frequencies of apoptotic (Figure 5C) and dead cells (Figure 5D) in CD4^+^ T cells from ART HIV-1^+^ compared with HIV^free^ individuals following incubation with rFasL alone, suggesting that ART HIV-1^+^ CD4^+^ T cells had an intrinsic higher predisposition to die by Fas-mediated apoptosis than HIV^free^ CD4^+^ T cells. We also noticed a synergistic effect in the induction of apoptotic cells (Figure 5C) and cell death (Figure 5D) when we treated purified CD4^+^ T cells with IMQ followed by rFAsL, when compared with cells incubated with rFasL alone. This effect was less pronounced in CD4^+^ T cells from HIV^free^ than from ART HIV-1^+^ individuals. We next analyzed *CASP8* mRNA levels by quantitative PCR (qPCR) and found a synergism of the stimulation with IMQ followed by rFasL in the induction of *CASP8* mRNA in CD4^+^ T cells from ART HIV-1^+^ donors only, when compared with rFasL alone (Figure 5E). Taken together, our results show that the TLR7/IRF-5 pathway feeds into the Fas/FasL signaling pathway, which subsequently amplifies IRF-5 and Caspase 8 expression, creating a spiral of death.

### IRF-5 inhibitory peptides limit IMQ- and Fas/FasL-induced cell death.

**Figure 6:**
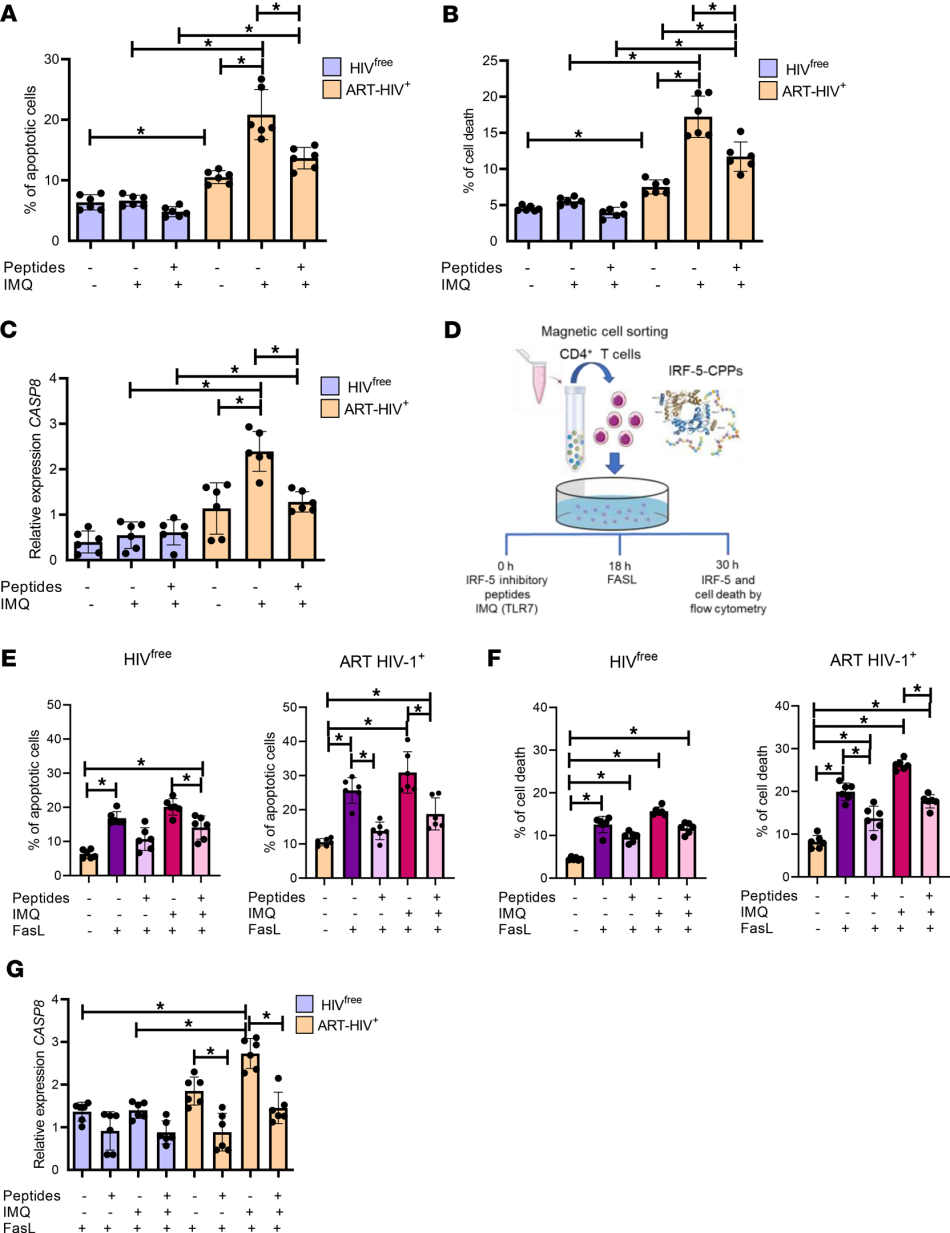
IRF-5 inhibitory peptides rescue memory CD4^+^ T cells from Fas-mediated apoptosis. (**A**–**C**) Purified CD4^+^ T cells from ART HIV-1^+^ and HIV^free^ individuals were pretreated with 10 μM of IRF-5–CPPs for 30 minutes before stimulation with IMQ and incubated at 37°C for 30 hours. Graphs show (**A**) the percentage of apoptotic cells, (**B**) the percentage of dead cells, and (**C**) *CASP8* mRNA expression levels in memory CD4^+^ T cells from ART HIV-1^+^ and HIV^free^ donors. (**D**) Experimental design. Purified CD4^+^ T cells from ART HIV-1^+^ and HIV^free^ individuals were pretreated with 10 μM of IRF-5–CPPs for 30 minutes, stimulated with or without IMQ for 12 hours, and incubated for a further 18 hours with or without rFasL. (**E**–**G**) Graphs show (**E**) the percentage of apoptotic cells, (**F**) the percentage of dead cells, and (**G**) *CASP8* mRNA expression levels in memory CD4^+^ T cells from ART HIV-1^+^ and HIV^free^ donors. Data are presented as the mean ± SD. The Friedman’s followed by the Dunn’s multiple-comparison tests were used for significance. *P < 0.05. *n* = 6.

Our findings so far indicate that IRF-5 is a key molecule in both the TLR7/IRF-5 axis and the Fas/FasL signaling pathway. Hence, we finally sought to block IRF-5 activation to limit cell death. To this end, we decided to use IRF-5 inhibitory peptides developed by Banga et al. (35). These cell-permeable peptides (IRF-5-CPPs) efficiently block IRF-5 function downstream of IRF-5 phosphorylation, preventing subsequent IRF-5 activation in various cell types (35); however, IRF-5–CPPs have yet to be tested in T cells. Thus, we chose 4 different peptides (P1–P4) with different cell specificities (Table 2). We first tested the cytotoxicity of different IRF-5–CPPs, including the control peptide, on human CD4^+^ T cells from HIV^free^ donors at concentrations varying from 0 μM to 50 μM. IRF-5–CPPs were found to be noncytotoxic up to 20 μM (Supplemental Figure 6A). When we tested the ability of IRF-5–CPPs to inhibit nuclear localization of human IRF-5 in untreated and IMQ-stimulated CD4^+^ T cells from ART HIV-1^+^ and HIV^free^ donors, we observed that P1 and P3 were the most effective peptides at preventing IRF-5 activation in unstimulated CD4^+^ T cells (Supplemental Figure 6B; about 62% and 60% inhibition following P1 and P3 treatment, respectively) as well as in IMQ-treated cells (Supplemental Figure 6C). We also tested whether P1 and P3 could be used together to achieve a stronger IRF-5 blockade and ultimately inhibit IMQ-induced cell death. To this end, CD4^+^ T cells from ART HIV-1^+^ and HIV^free^ individuals were treated with either 5 μM P1 or P3 alone, or with a mixture of P1 and P3, and subsequently stimulated with IMQ. As shown in Supplemental Figure 6D, the P1/P3 mix was slightly more efficient in blocking IRF-5 activation in ART HIV-1^+^ CD4^+^ T cells upon stimulation with IMQ. Indeed, we observed 44% inhibition of IRF-5 nuclear localization following treatment with P1, 40% after incubation with P3, and about 60% when cells were cocultured with P1 and P3 together. Thus, we decided to use a combination of P1 and P3 to inhibit the TLR7/IRF-5 pathway; the control peptide was used for all “Peptide^neg^” control groups. To this end, we treated purified CD4^+^ T cells with the peptide mix for 30 minutes before stimulation with IMQ. The combination of the 2 IRF-5–CPPs was able to significantly block apoptosis in memory CD4^+^ T cells from ART HIV-1^+^ individuals. Indeed, we observed lower frequencies of apoptotic (Figure 6A) and dead (Figure 6B) cells in memory CD4^+^ T cells from ART HIV-1^+^ individuals following IMQ stimulation. As expected, this was not the case in HIV^free^ donors, since these cells express very low levels of TLR7 and do not respond to IMQ (Figure 3, A, C, and D). Similar results were obtained when we analyzed *CASP8* mRNA levels, which were significantly reduced when we added IRF-5 blocking peptides to purified CD4^+^ T cells from ART HIV-1^+^ individuals stimulated with IMQ (Figure 6C).

Since the Fas/FasL pathway is involved in memory CD4^+^ T cell death in HIV-1 infection (9), we next assessed whether the IRF-5–CPP mix could block Fas-mediated apoptosis in memory CD4^+^ T cells from ART HIV-1^+^ individuals. CD4^+^ T cells were incubated for 1 hour with 5 μM of a mixture of P1 and P3 or the control peptide, followed by an 18-hour incubation with or without IMQ. Cells were then treated with rFasL for another 12 hours (Figure 6D). Remarkably, the IRF-5–CPP mix could significantly limit Fas-mediated apoptosis. In fact, the increase in the frequency of apoptotic cells (Figure 6E) or dead cells (Figure 6F) following rFasL or IMQ + rFasL treatment was approximately reduced by half after IRF-5 blockade in CD4^+^ T cells from ART HIV-1^+^ donors. A similar but less pronounced effect was observed in CD4^+^ T cells from HIV^free^ individuals. No differences were observed between CD4^+^ T cells from male and female HIV^+^ or HIV^free^ donors (Supplemental Figure 7, A–D).

Finally, to clearly prove that inhibition of Fas-mediated apoptosis was indeed dependent on IRF-5 blockade, we assessed *CASP8* mRNA levels after the various treatments; Caspase 8 is a downstream molecular target of IRF-5 (22). We observed a nearly 50% reduction in the induction of *CASP8* mRNA in CD4^+^ T cells from ART HIV-1^+^ donors treated with either rFasL or IMQ + rFasL following IRF-5 blockade (Figure 6G). The effect in memory CD4^+^ T cells from HIV^free^ samples was only partial and less pronounced than observed in ART HIV-1^+^CD4^+^ T cells (Figure 6G). We did not notice any difference between CD4^+^ T cells from male and female donors (Supplemental Figure 7E). Taken together, our results suggest that IRF-5 plays a central role in ART HIV-1^+^ memory CD4^+^ T cell death, not only as a downstream target of TLR7 but also directly involved in Fas-mediated apoptosis.

### IFN-β and DAMPs promote TLR7 expression in CD4^+^ T cells.

**Figure 7:**
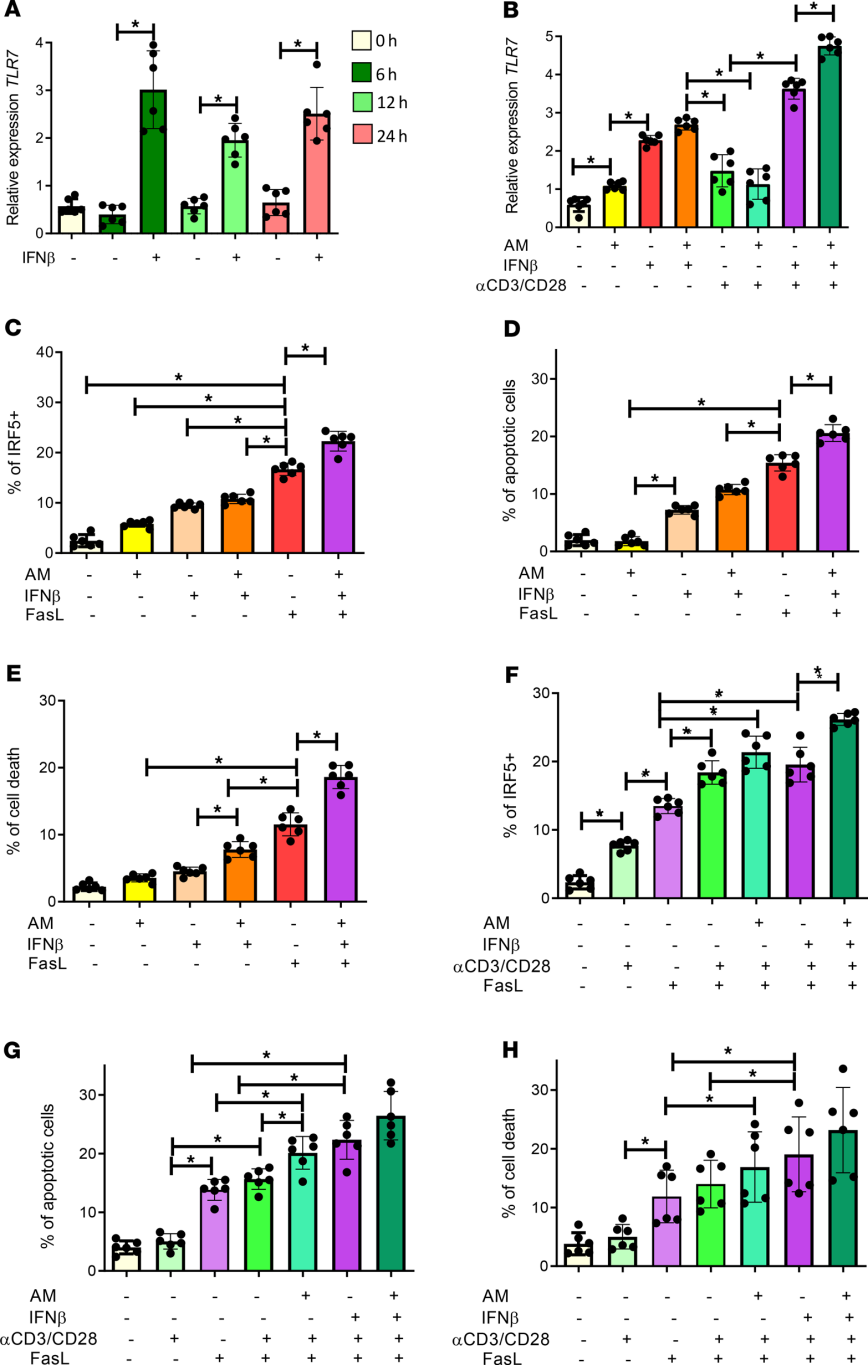
IFN-β and DAMPs promote TLR7 expression on CD4^+^ T cells. (**A**) Purified CD4^+^ T cells from HIV^free^ individuals were incubated with IFN-β or with medium alone. Graph shows RT-PCR analysis of *TLR7* expression at 0, 6, 12, and 24 hours of treatment. (**B**) Purified CD4^+^ T cells from HIV^free^ individuals were treated with IFN-β in the presence or absence of conditioned medium with 10% v/v apoptotic material (AM, supernatant of staurosporine-treated PBMCs) for 24 hours, before stimulation with αCD3α/CD28 for a further 24 hours. Graphs shows RT-PCR analysis of *TLR7* gene expression. (**C**–**E**) Purified CD4^+^ T cells from HIV^free^ individuals were incubated with IFN-β in the presence or absence of conditioned medium with 10% v/v apoptotic material for 24 hours, before adding rFasL for a further 18 hours. Graphs represent (**C**) the percentage of IRF-5^+^, (**D**) the percentage of apoptotic cells, and (**E**) the percentage of dead CD4^+^ T cells after incubation with the indicated culture conditions. (**F**–**H**) Purified CD4^+^ T cells from HIV^free^ donors were treated with IFN-β in the presence or absence of conditioned medium with 10% v/v apoptotic material for 24 hours; they were then stimulated with αCD3/αCD28 for a further 24 hours and finally incubated for 18 hours with rFasL. Graphs represent (**F**) the percentage of IRF5^+^, (**G**) the percentage of apoptotic cells, and (**H**) the percentage of dead CD4^+^ T cells after incubation as described above. Data are presented as the mean ± SD. The Friedman’s followed by the Dunn’s multiple-comparison tests were used for significance. *P < 0.05. *n* =6.

Although TLR7 expression on T cells has been reported in mice and humans (28, 36–38), the signaling pathways promoting TLR7 upregulation in CD4^+^ T cells remain unknown. Type I IFN was shown to enhance TLR expression in B cells (39–42). Since IFN-β is often part of chronic inflammatory environments associated with persistent infections (43) and since TLR7 is upregulated in CD4^+^ T cells during persistent infections (13), we first assessed whether purified CD4^+^ T cells from HIV^free^ individuals exposed to recombinant IFN-β would upregulate TLR7. We found that IFN-β promoted *TLR7* mRNA upregulation as early as 6 hours after initial exposure and that this expression was maintained over 24 hours (Figure 7A). Tissue disruption and release of DAMPs are also hallmarks of chronic inflammatory environments. Thus, we next exposed CD4^+^ T cells from HIV^free^ donors to apoptotic cell material. Interestingly, apoptotic cell material could also enhance *TLR7* mRNA expression, although to a lesser extent than IFN-β; nevertheless, its effect was synergistic with IFN-β (Figure 7B). This suggests that CD4^+^ T cells in an inflammatory environment can upregulate TLR7 independently from their antigen specificity. Following this, we assessed whether CD4^+^ T cell priming in the presence of DAMPs and IFN-β can modulate *TLR7* mRNA expression levels. Stimulation with anti-CD3/CD28 in the presence or absence of apoptotic cell material slightly induced *TLR7* expression (Figure 7B). However, anti-CD3/CD28 stimulation of cells previously exposed to IFN-β greatly promoted *TLR7* expression, which was further enhanced in the presence of DAMPs (Figure 7B), indicating that antigen specific CD4^+^ T cells primed in a persistent inflammatory environment rich in IFN-β and DAMPs are bound to express high levels of *TLR7*.

To test whether CD4^+^ T cells exposed to IFN-β and/or apoptotic cell material with or without anti-CD3/CD28 stimulation were more susceptible to Fas-mediated apoptosis, we treated cells with rFasL. When we analyzed memory CD4^+^ T cells, we found higher frequencies of IRF-5^+^ (Figure 7C), apoptotic (Figure 7D), and dead cells (Figure 7E) when CD4^+^ T cells were exposed to IFN-β + DAMPs prior to rFasL treatment, compared with rFasL alone. Anti-CD3/CD28 stimulation did not alter the cells’ susceptibility to Fas-mediated apoptosis (Figure 7, G and H) but slightly increased IRF-5 expression (Figure 7F). In agreement with the *TLR7* expression levels shown in Figure 7B, CD4^+^ T cells exposed to IFN-β + DAMPs prior to anti-CD3/CD28 stimulation were significantly more susceptible to Fas-mediated apoptosis (Figure 7, G and H) and expressed the highest levels of IRF-5 (Figure 7F) of any treatment group. Similar results were obtained when we analyzed memory CD4^+^ T cells (Supplemental Figure 8, A–F). Taken together, these results suggest that exposure of antigen-specific or bystander CD4^+^ T cells to apoptotic material and IFN-β promotes the upregulation of TLR7 and predisposes cells to FAS-mediated cell death.

**Figure 8:**
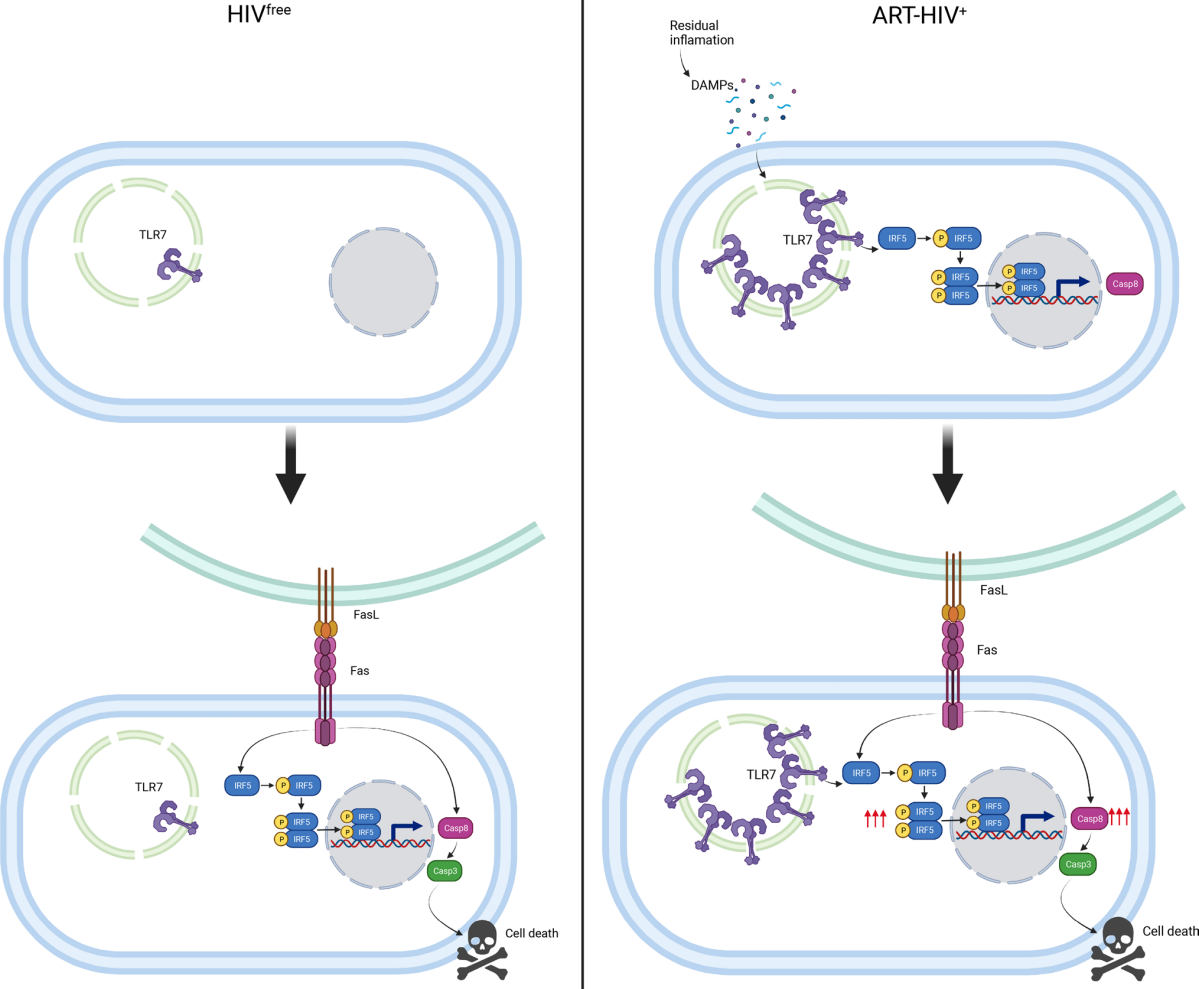
Proposed model. Residual inflammation maintains the upregulation of TLR7 and IRF-5 in memory CD4^+^ T cells from ART HIV-1^+^ individuals but not in HIV^free^ individuals. The activation of the TLR7/IRF-5 axis feeds into the Fas/FasL pathway, predisposing and enhancing Fas-mediated apoptosis only in memory CD4^+^ T cells from ART HIV-1^+^ individuals. Finally, we proposed that this mechanism can be blocked by adding IRF-5 inhibitory peptides, which block the predisposition to Fas-mediated cell death. Created with BioRender.com.

**Figure 9:**
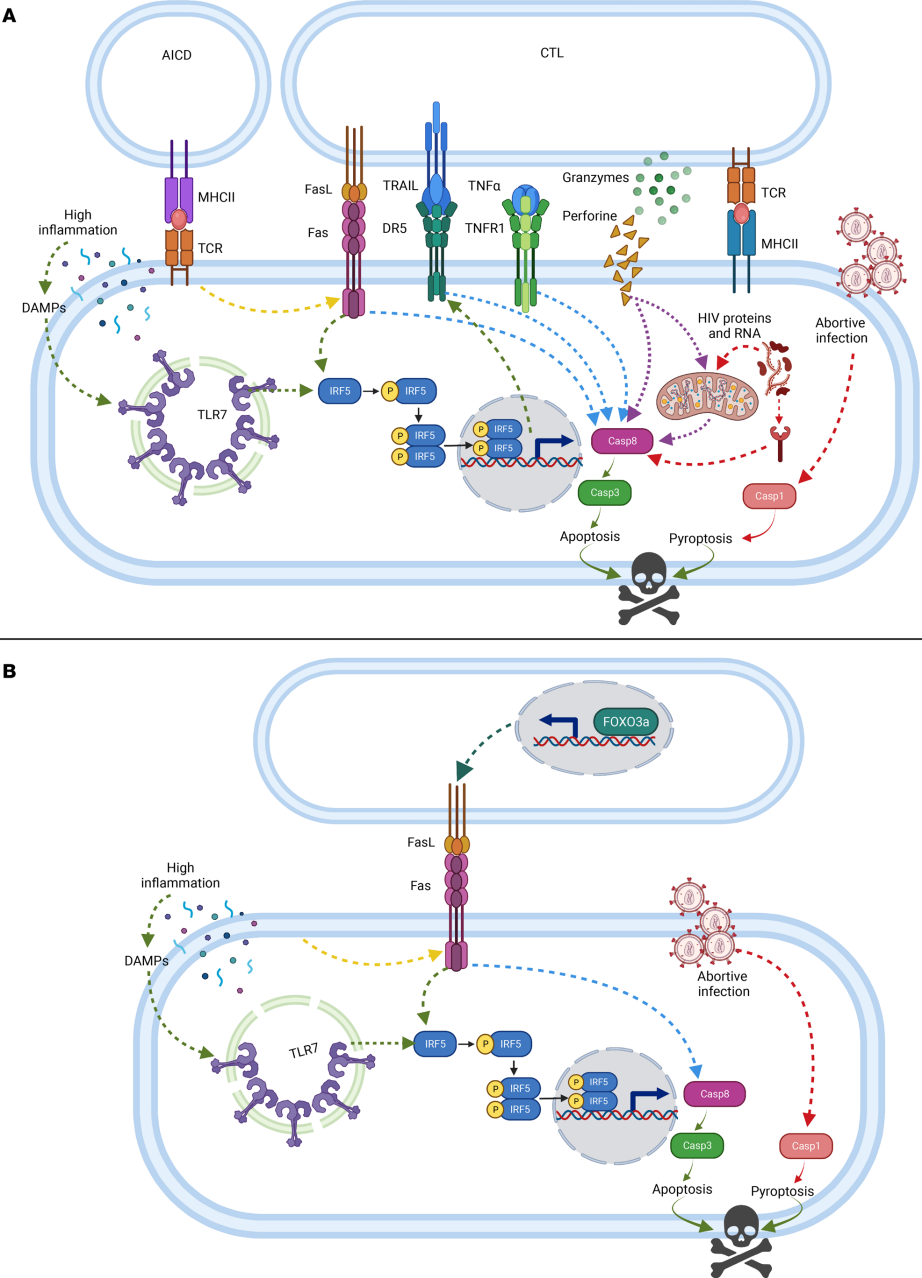
Summary of cell death pathways occurring in CD4^+^ T cells during primary HIV-1 infection or following ART. (**A**) A large proportion of CD4^+^ T cell death during primary HIV-1 infection occurs via apoptosis and comprises extrinsic and intrinsic pathways. Extrinsic apoptosis involves death receptor members of the tumor necrosis factor receptor gene superfamily, such as Fas, TNFR1, and DR5 (TRAILR2). The activation of these receptors by a death ligand results in the activation of Caspase 8. We propose that the TLR7/IRF-5 pathway feeds into these pathways by sensitizing cells to Fas-, TNFR1-, and DR5-mediated apoptosis through the induction of Caspase 8 and IRF-5. Other extrinsic cell death pathways include activation-induced cell death (AICD) and killing by cytotoxic CD8^+^ T cells (CTL) via secretion of perforin and granzymes. Intrinsic apoptosis includes activation of Caspase 3 by HIV proteins and DNA. Finally, abortively infected CD4^+^ T cells die by pyroptosis, a highly inflammatory form of programmed cell death that results from the activation of Caspase 1. (**B**) Although antiretroviral therapy restores CD4^+^ T cell counts, memory CD4^+^ T cells from ART HIV-1^+^ individuals are still prone to apoptosis, despite treatment. These cells mostly die by Fas-mediated apoptosis. The Fas/FasL pathway is strengthened on one hand by the transcription factor FOXO3a, which empowers the “killers,” and, on the other hand, by the TLR7/IRF-5 axis, which weakens the “victims.” Death by pyroptosis in abortively infected cells may also occur. Created with BioRender.com.

In summary, we propose that memory CD4^+^ T cells from ART HIV-1^+^ individuals express TLR7 and IRF-5, which represent an imprint that predisposes these cells to Fas-mediated apoptosis. The TLR7/IRF-5 axis can be activated by DAMPs, resulting in heightened Caspase 8 expression. This pathway directly feeds into the Fas-FasL pathway, which increases IRF-5 and Caspase 8 expression, leading to cell death. In contrast, memory CD4^+^ T cells from HIV^free^ individuals and from ECs express low levels of TLR7 and IRF-5, which makes these cells less capable of sensing DAMPs and ultimately less prone to Fas-mediated apoptosis (Figure 8). Figure 9 summarizes how this pathway could work synergistically with other known cell death pathways (reviewed by refs.44, 45), particularly those requiring Caspase 8, to induce apoptosis in CD4^+^ T cells during PHI and after ART.

## Discussion

Although most PLWH on ART recover CD4^+^ T cell counts, it remains unclear why memory CD4^+^ T cells are highly sensitive to apoptosis. Here we show that memory CD4^+^ T cells from HIV^+^ donors undergoing ART display an imprint by expressing higher levels of *TLR7* mRNA. We propose that this allows memory CD4^+^ T cells to sense inflammatory tissue damage–derived DAMPs and activates the TLR7/IRF-5 pathway, making cells more susceptible to Fas-mediated apoptosis. Treatment with IRF-5 blocking peptides successfully blocks the TLR7/IRF-5 pathway and significantly reduces memory cell death by Fas-mediated apoptosis.

PLWH on ART are characterized by inflammation and express on CD4^+^ T cells higher levels immune checkpoint molecules like PD-1, CTLA-4, LAG-3, and TIGIT compared with HIV^free^ participants (46). In addition to their exhaustion function, these molecules contribute to the establishment and maintenance of a latent cellular infection (46–49). Our findings show that memory CD4^+^ T cells from patients on ART also differed from those from HIV^free^ individuals and EC in their TLR7 expression. Following TLR7 triggering, IRF-5 was activated and promoted Caspase 8 in memory CD4^+^ T cells from ART HIV-1^+^ participants. A significant proportion of memory and effector CD4^+^ T cells expressed active IRF-5 and upregulated *CASP8*, suggesting that the TLR7/IRF-5 axis was active in some cells. Still, the factors that activate TLR7 in CD4^+^ T cells in ART treated PLWH remain to be unveiled.

In our previous work, we have shown that CD4^+^ T cells are capable of sensing inflammatory tissue damage–derived DAMPs via TLR7 in a murine model of visceral leishmaniasis (13). Since patients on ART display residual levels of immune activation and inflammation, it is possible that inflammation could be involved in generating DAMPs derived from tissue damage occurring at the intestinal mucosal site (50, 51), a major HIV reservoir. The fact that IRF-5 is highly expressed and active in CD4^+^ T cells from PHI suggests that the level of inflammation, and consequently DAMPs release, is proportional to IRF-5 upregulation. Nevertheless, activation of the TLR7/IRF-5 axis in CD4^+^ T cells may be multifactorial and could also be triggered by microbial products released into the circulation from the gut lumen due to compromised gastrointestinal mucosa (50). An additional source of DAMPs could be derived from material released by nonpermissive, resting CD4^+^ T cells dying by pyroptosis following abortive HIV infection (4, 5). Moreover, HIV-1 is an enveloped retrovirus with 2 copies of a ssRNA genome that may be recognized by TLR7 (52, 53). To this end, it would be interesting to analyze IRF-5 expression in CD4^+^ T cells located at anatomical reservoir sites, like in the gut mucosa.

Independently from the agonist molecule that triggers TLR7, ART-treated PLWH are characterized by an imprint on CD4^+^ T cells, likely occurring before ART, that makes them more susceptible to sensing DAMPs, HIV, or both. This imprint is also responsible for the activation of IRF-5 and Caspase 8 and for sensitizing memory cells to Fas-mediated apoptosis. Interestingly, we show that TLR7 and IRF-5 expression levels directly correlate with CD4^+^ T cell death in PLWH on ART. IRF-5 expression is also inversely correlated with CD4^+^ T cell counts in PHI, suggesting that this pathway may also operate early during acute infection and could lead to the death of bystander CD4^+^ T cells. Hence, activation of the TLR7/IRF-5 axis and subsequent predisposition to Fas-mediated apoptosis might also affect CD4^+^ T cell homeostasis in general and might not be solely restricted to the memory compartment. Thus, it would be interesting to investigate the role of IRF-5 in patients with HIV-1^+^ who do not respond to therapy; these patients are often referred to as immune nonresponders (54). Incomplete CD4^+^ T cell recovery (below 350 cells, despite years on effective ART) has been associated with increased replicative senescence, an increase in effector CD4, and higher sensitivity to cell death, all of which contribute to a lower proliferative capacity of CD4^+^ T cells (6, 55–58). More importantly, the extent of lymphoid tissue damage has been shown to influence CD4^+^ T cell count reconstitution (59), suggesting that the level of inflammation and the extent of inflammatory tissue damage at the time of therapy affects CD4^+^ T cell recovery. Tissue disruption results in the release of DAMPs, which could trigger TLR7 expressed on CD4^+^ T cells and activate the TLR7/IRF-5 axis. Therefore, IRF-5 blockade could be beneficial in PLWH that fail to recover or show a slow recovery of CD4^+^ T cells after ART.

Of note, despite having an active infection and higher levels of systemic immune activation compared with HIV^free^ individuals (60), CD4^+^ T cells from EC donor expressed low levels of TLR7, insufficient to promote IRF-5 and cell death.

TLR7 agonists, in combination with other therapies, have been shown to promote sustained control of simian HIV (SHIV) or simian immunodeficiency virus (SIV) in nonhuman primates (61–66). Indeed, the TLR7 agonist GS-9620 was shown to activate HIV in PBMCs from ART HIV^+^ individuals and to reduce viral replication via the production of IFN-α (67, 68). However, only modest effects were observed in HIV controllers on ART who received an oral TLR7 agonist (69). The mild effect was to be attributed to increased DC and NK cell crosstalk and an increase in cytotoxicity potential (69). Similarly to ECs, rhesus macaques are capable of naturally controlling SIV infection. It is, thus, possible that their CD4^+^ T cells do not express high levels of TLR7. In this case, the use of TLR7 agonists will not affect the memory CD4^+^ T cell compartment. However, our results suggest that the therapeutic use of TLR7 agonists could lead to an imbalance in CD4^+^ T cell homeostasis and a loss of memory and effector CD4^+^ T cells in PLWH on ART with CD4^+^ T cells expressing high TLR7 levels.

Several important questions arose from our study: (a) What induces TLR7? (b) What determines TLR7 expression levels in CD4^+^ T cells? (c) Are TLR7 expression levels associated with the capacity to maintain CD4^+^ T cell homeostasis during primary infection?

We tried to identify possible pathways that could lead to TLR7 upregulation in CD4^+^ T cells and found that IFN-β together with DAMPs display a synergistic effect in inducing TLR7 expression in CD4^+^ T cells. Although this possible synergism can be enhanced by TCR triggering, TCR engagement is not strictly required. These results imply that the level of IFN-I response and inflammation may determine the level of TLR7 with which CD4^+^ T cells (antigen-specific and bystanders) will be imprinted. It is largely recognized that TLR7 expression in innate immune cells, particularly plasmacytoid DCs (pDCs), is essential for inducing the IFN-I response, which is required to control HIV spread during the acute phase of infection (70, 71). However, IFN-I may also have immunosuppressive effects in HIV infection (72, 73). Hence, a balance between the double-edge IFN-I functions, antiviral and immune suppression, is crucial for the outcome of HIV infection (74). A possible explanation for variations in IFN-I levels among different individuals could be given by the existence of TLR7 polymorphisms (75–80), which could determine the amount of IFN-I that is produced (79). Polymorphisms in the *IRF5* gene could also explain why the TLR7/IRF-5 axis is more activated in certain individuals than others. IRF5 polymorphisms have been well described for autoimmune disease and are often associated with various levels of inflammation (81–84).

Our findings highlight the central role played by IRF-5, which is not only involved in predisposing memory CD4^+^ T cells to Fas-mediated apoptosis, but is also a key component of the Fas/FasL pathway. The IRF-5 effects are complementary to Foxo3a, which enhances the expression of FasL (9, 85), empowering the “killers,” while IRF-5 promotes the expression of Fas (22), weakening the “victims.” It is thus not surprising that IRF-5 blockade limited not only the induction of Caspase 8 via the TLR7/IRF-5 axis but also Fas-mediated cell death. IRF-5 blockade could be developed as a possible therapy to prevent the loss of protective memory CD4^+^ T cells in patients under ART and to help recover CD4^+^ T cell counts in patients with HIV^+^ who do not respond to therapy. Since a heightened expression of IFN-I and tissue damage are common features of persistent inflammatory responses, it is possible that the pathway we describe in this study is not only involved in HIV-1-pathogenesis but could also operate during other chronic viral infections, such as SARS-CoV-2.

In conclusion, we report that CD4^+^ T cells from PLWH receiving ART display heightened *TLR7*, IRF-5, and *CASP8* expressions compared with CD4^+^ T cells from EC and HIV^free^ donors. Moreover, activation of the TLR7/IRF-5 axis feeds into the Fas/FasL pathway predisposing and enhancing Fas-mediated apoptosis. IRF-5 plays a central role in both pathways, and blockade of IRF-5 activation limits cell death. We propose IRF-5 blockade as a possible therapeutic target to prevent memory CD4^+^ T cell loss in patients under ART and, more importantly, for patients who have a low CD4^+^ T cell recovery despite long-term ART that represent 20% of patients with a persistent risk to develop non-AIDS and AIDS comorbidities.

## Methods

### Study population.

Participants (27–56 years old) were selected from the Montreal Primary HIV infection study. Age-matched uninfected participants were selected as negative control for HIV-1 infection. PLWH included in the study displayed similar clinical and virological data. PHI participants, whose viral acquisition was estimated to have occurred less than 4 months prior to sample collection displayed viral loads ranging from 3.08 ***×*** 10^3^ to 513.54 ***×*** 10^3^ copies/mL. PLWH on ART displayed both viral suppression (viral loads < 40 copies/mL) and CD4^+^ T cell recovery (>400 cells/μL in blood after treatment) for at least 5 years. EC participants displayed a long-term viral control (viral loads < 40 copies/mL) without treatment for > 5 years and did not have protective gene polymorphisms such as CCR5 Δ32 and B57/B27 haplotypes, with blood CD4^+^ T cell counts > 400 cells/μL. Demographic and clinical characteristics of each study group are summarized in Table 1.

### Cell culture reagents.

Cells were cultured in RPMI 1640 (Invitrogen) supplemented with 3.2 % L-Glutamine (Cellgro), 2-mercaptoethanol, sodium pyruvate, penicillin/streptomycin (Invitrogen), and 10% FBS (Wisent). The antiviral Azidothymidine (AZT, Sigma-Aldrich; 10 μM) was added to all cultures from PLWH to prevent viral replication and de novo infections, as previously reported (86). Cells were washed with Dulbecco’s PBS (Invitrogen) before RNA extraction or immunofluorescence staining for flow cytometry.

### CD4^+^ T cell purification.

PBMC were thawed, resuspended in 1 mL of FBS, and centrifuged for 5 minutes at 300*g* at room temperature. Pelleted cells were resuspended in 3 mL RPMI with 10% FBS and incubated at 37°C for 2 hours before CD4^+^ T cell purification.

CD4^+^ T cells from ART HIV-1^+^, EC, and HIV^free^ samples were isolated from PBMC using the EasySep human CD4^+^ T cell enrichment kit (StemCell Technologies) following manufacturer instructions, allowing for > 92% purity.

### In vitro CD4^+^ T cell stimulation.

In total, 1 ***×*** 10^6^ CD4^+^ T cells were activated for 24 hours with 1 μg/mL of anti-CD3 (clone OKT3, eBioscience) and 1 μg/mL of anti-CD28 Abs (clone CD28.2, eBioscience). To assess TLR7 agonist effect on CD4^+^ T cells, 1 ***×*** 10^6^ cells/mL were stimulated with 5 μg/mL of IMQ (Invivogen) for 30 hours. For IFN-β stimulation, CD4^+^ T cells were cultured with 10 ng/mL IFN-β (Peprotech) for 6 hours and washed 3 times with RPMI. For some assays, 1 μg/mL of Brefeldin A (BD Biosciences) was added to cell cultures for 6 hours to evaluate the intracellular accumulation of inflammatory cytokines.

To investigate pathways promoting TLR7 expression in CD4^+^ T cells, we pretreated 1 ***×*** 10^6^ HIV^free^ CD4^+^ T cells with 10 ng/mL IFN-β for 6 hours in the presence or absence of conditioned medium with 10% v/v of DAMPs containing supernatant, obtained from PBMCs treated with staurosporine for 24 hours, before TCR activation or TLR7 stimulation. The supernatant was prepared as follows: 1 ***×*** 10^6^/mL PBMC from HIV^free^ donors were cultured in vitro in the presence of 2 μM staurosporine for 2 hours at 37°C; cells were then washed 3 times with PBS and left in culture for a further 4 hours at 37°C, before collecting the supernatant.

### Fas-induced apoptosis and cell death.

Purified CD4^+^ T cells were cultured in the presence or absence of 10 ng/mL rFasL (BioLegend) for 18 hours. Apoptosis detection was performed using rabbit anti–human cleaved Caspase-3 (Asp175) PE (clone D3E9, Cell Signaling Technology), annexin V-APC (BD Biosciences or BioLegend), and Zombie Aqua Fixable Viability Kit (BioLegend) by flow cytometry. The percentage of Fas-induced apoptosis was determined by the formula: percentage of apoptosis in the presence of rFasL minus percentage of apoptosis in absence of rFasL, as previously described (9).

### CD4^+^ T cells and PBMC stimulation in the presence of inhibitory peptides.

We first analyzed the cytotoxicity of different IRF-5–CPPs, described in Table 2. Cells were incubated with various concentrations of IRF-5–CPPs (5, 20, and 50 μM) for 24 hours. We evaluated cell death by flow cytometry and determined IRF-5 nuclear localization after IMQ treatment by ImageStreamX. A scrambled version of the peptides was used as a negative control. CD4^+^ T cells were pretreated with 10 μM of IRF-5–CPPs for 30 minutes before stimulation with 5 ng/mL IMQ for 30 hours at 37°C, or pretreated cells were stimulated with 5 ng/mL IMQ, and 12 hours later, 10 ng/mL rFasL was added to the culture for a further 18 hours of incubation.

### Flow cytometry.

Purified CD4^+^ T cells and PBMC were labeled with mouse anti–hCD3-BV711 (clone UCHT1, BD Biosciences or BioLegend), hCD4-Alexa Fluor 700 (clone RPA-T4, BD Biosciences or BioLegend), hCD45RA-APC-H7 (clone HI100, BD), hCD45RA-APC-Cy7 (clone HI100, BioLegend), hCD27-PerCP-Cy5.5 (clone RPA-T4, BD Biosciences or BioLegend), hCD197 (CCR7) BV605 (clone 2-L1-A, BD), hCD197 (CCR7) BV605 (clone G043H7, BioLegend), hIFN-γ–PE-Cy7 (clone 4S.B3, BD), hDR5 (CD262) (clone B-K29, BD), CD95-FITC (clone DX2, BD), hCD95-PE (clone DX2, BD), hCD95-PE-CF594 (clone DX2, BD), hIgG1 κ isotype control PE-CF594 (clone X40, BD Biosciences), and hIgG1 κ isotype control–PE-Cy7 (clone MOPC-21, BD Biosciences); rabbit anti–hIRF-5–PE (clone E1N9G, Cell Signaling Technology, discontinued), human cleaved Caspase-3 (Asp175) PE (clone D3E9, Cell Signaling Technology), annexin V APC (BD Biosciences and BioLegend), and hIgG XP-PE isotype control (clone DA1E, Cell Signaling Technology); sheep anti–hIRF-5–Alexa Fluor 488 (IC4508G, R&D systems) and hIgG isotype control-Alexa Fluor 488 (IC016G, R&D systems); and Zombie Aqua Fixable Viability Kit (BioLegend). Cells were fixed with 4% paraformaldehyde for 10 minutes at room temperature, permeabilized with 0.25% (w/v) saponin in PBS, and stained intracellularly with rabbit anti–hIRF-5 PE (clone E1N9G, Cell Signaling Technology), sheep anti–hIRF-5–Alexa Fluor 488 (R&D Systems), rabbit anti–human Cleaved Caspase-3 (Asp175) PE (clone D3E9, Cell Signaling Technology), mouse anti-hIgG1 κ isotype control PE-CF594 (clone X40, BD Biosciences), mouse anti–hIgG1 κ isotype control PE-Cy7 (clone MOPC-21, BD Biosciences), rabbit anti–hIgG XP-PE isotype control (clone DA1E, Cell Signaling Technology), and sheep anti-hIgG isotype control Alexa Fluor 488 (R&D Systems). Flow cytometry analysis was performed with a BD LSRII flow cytometer (BD Biosciences). Data were analyzed with FlowJo and DIVA software (BD Biosciences). In total, 200,000–500,000 gated cells were analyzed for each sample.

### ImageStreamX flow cytometry.

Samples were stained with mouse anti-hCD3-BV711 (clone UCHT1, BD and BioLegend), hCD4-Alexa Fluor 700 (clone RPA-T4, BD Biosciences and BioLegend), hCD8-BV650 (clone RPA-T8, BD Biosciences), hCD19-V450 (clone HIB19, BD Biosciences), and hCD14 PE-Cy7 (clone M5E2, BD Biosciences). The cells were then fixed, permeabilized, and stained with DAPI (Invitrogen) to visualize the nucleus and mouse anti–hIFN-γ PE-Cy7 (clone 4S.B3, BD Biosciences), along with rabbit anti–hIRF-5 PE (clone E1N9G, Cell Signaling Technology) or sheep anti–hIRF-5 Alexa Fluor 488 (R&D Systems). Finally, samples were acquired using the ImageStreamX MKII flow cytometer and analyzed with IDEAS software (Amnis). The localization wizard was used to determine the nuclear localization of IRF-5. The morphology mask was used to determine a similarity score, which quantifies the correlation of pixel values of the DAPI and IRF-5 images on a per-cell basis. A similarity score > 1 was used as a cut-off for nuclear localization. Cells in individual bins were visually inspected to confirm subcellular localization (values < or > 1). In total, 200,000–500,000 gated cells were analyzed for each sample.

### RNA isolation and qPCR analysis.

RNeasy mini kit (QIAGEN) was used for RNA isolation. RNA yield and purity were measured by NanoDrop (Thermo Fisher Scientific). cDNAs were generated by reverse transcription using the iScript cDNA synthesis kit (Bio-Rad). Real-time PCR analysis was performed with the Stratagene mx3005p real-time PCR system using iTaq Universal SYBR Green Supermix (Bio-Rad) and standard cycle of amplification. *IRF5*, *TLR7*, *CASP8*, and *GAPDH* were amplified using the specific primers summarized in Table 3. GAPDH was used as a housekeeping gene to normalize the expression levels of target genes. Relative gene expression of target genes in each sample was determined by the comparative CT method (2^−ΔΔCt^) (87, 88).

### Generation of IRF5^–/–^ THP-1 cells.

The sgRNA for IRF-5 was obtained from IDT (target sequence 5′-GTA CTG GCA GCT GTT CAC CT-3′). Recombinant Alt-R S.p. Cas9 nuclease (85 μg) was incubated with each target sgRNA (6 μL of 100 μM stock) for 20 minutes at room temperature. The reaction was supplemented with Alt-R Cas9 Electroporation Enhancer (4 μL) and combined with 4 ***×*** 10^6^ THP-1 (ATCC) resuspended in complete P3 nucleofection solution (71 μL) (Lonza). The samples were subjected to nucleofection using program CM-137 and maintained in RPMI. At 5 days after nucleofection, 3 ***×*** 10^6^ cells were collected to confirm KO efficiency by immunoblot analysis.

### Statistics.

Parameters of patients and controls are described as absolute frequencies in the case of qualitative variables; as medians and IQRs for nonnormally distributed quantitative variables (age, viral loads, CD4 counts); and as mean ± SD for normally distributed quantitative variables. Comparison of quantitative variables of each study group of individuals and of frequencies, median fluorescence intensity, and cell numbers positive for each marker were performed using the Mann-Whitney *U* test (2 groups) and the Kruskal-Wallis test, followed by the Dunn’s multiple-comparison test (>2 groups). Variables of in vitro experiments performed with and without were compared with the Friedman’s test and the Dunn’s multiple-comparison test. Correlation analyses were performed with Pearson’s or Spearman’s coefficients. In all cases, *P* ≤ 0.05 was considered statistically significant. Statistical analyses were run in GraphPad Prism version 6 (GraphPad Software).

### Study approval.

Studies were approved by the McGill University Health Centre Ethical Review Board (SL-00.069) and the Ethics Committee for Research of INRS (CER-21-599). Written informed consent was obtained from all participants.

## Author contributions

LCP and S. Stäger conceived the project, designed the experimental approach, interpreted data, and wrote the manuscript with comments from all authors; LCP performed experiments and analyzed data; XDL, LTM, TS, and S. Swaminathan performed experiments; MRR, SI, and BJB provided key reagents and expertise; and JVG and JPR provided key expertise, interpreted data, and revised the manuscript.

## Supplementary Material

Supplemental data

## Figures and Tables

**Table 3:**
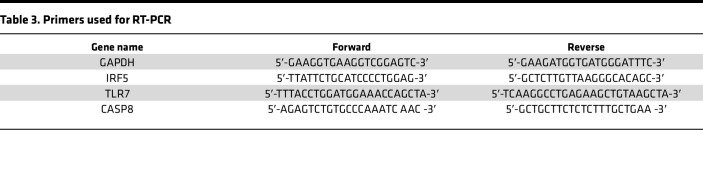
Primers used for RT-PCR

**Table 1:**
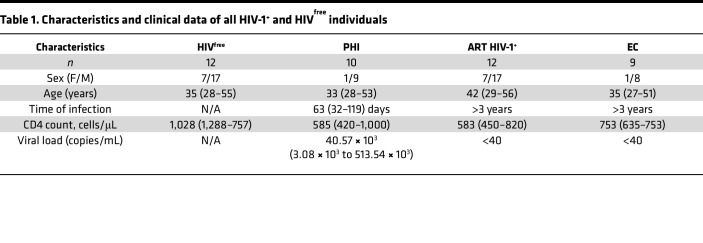
Characteristics and clinical data of all HIV-1^+^ and HIV^free^ individuals

**Table 2:**
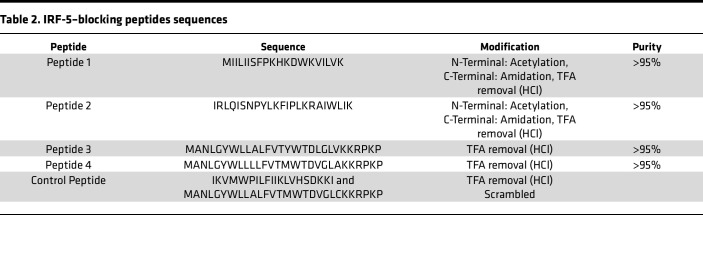
IRF-5–blocking peptides sequences

## Version 1 05/25/2023

In-Press Preview

## Version 2 07/10/2023

Electronic publication
