# Supplementary material for: The TLR7/IRF-5 axis sensitizes memory CD4+ T cells to Fas-mediated apoptosis during HIV-1 infection
Source: JCI Insight. 2023 Jul 10;8(13):e167329. doi: 10.1172/jci.insight.167329 (PMC10371351; doi:10.1172/jci.insight.167329)
Supplement: Supplemental data [file jciinsight-8-167329-s206.pdf]

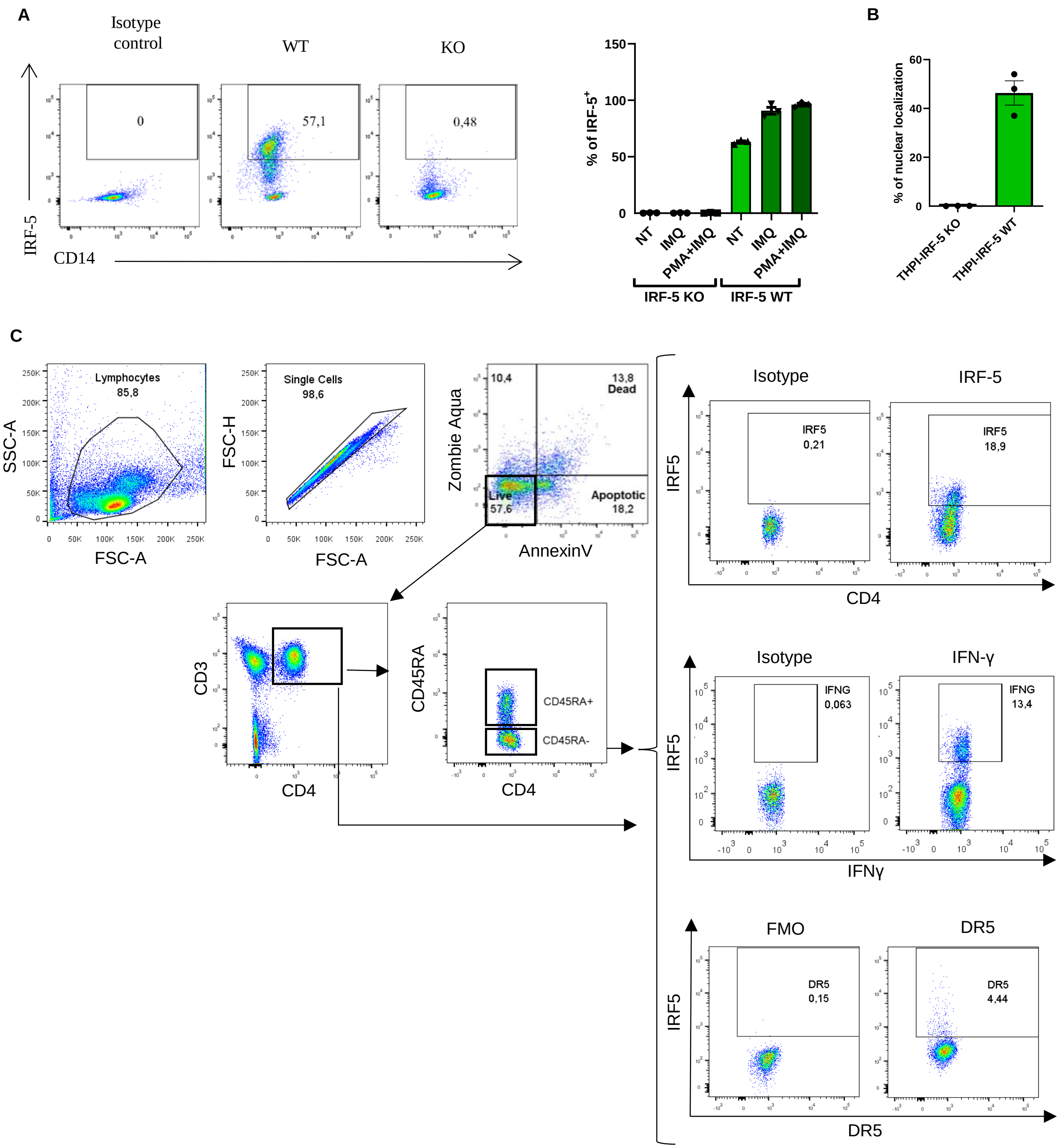

Supplemental Figure 1: Anti-IRF-5 antibody specificity and gating strategy. a) WT and *Irf5*$^{-/-}$ THP-1 cells were restimulated with either IMQ or PMA+IMQ and IRF-5 expression was assessed by flow cytometry. Graphs show a) representative FACS plots and the percentage of IRF-5 positive cells, and b) the frequency of IRF-5 nuclear localization. c) Representative FACS plots showing the gating strategy used to determine the frequency of cells positive for Annexin V, IRF-5^{+}, IFNɣ$^{+}$, and DR5^{+} in total CD4^{+} T cells or CD45RA^{+} and CD45RA^{-} CD4^{+} T cells in PBMCs isolated from PHI and HIV^{free} individuals.

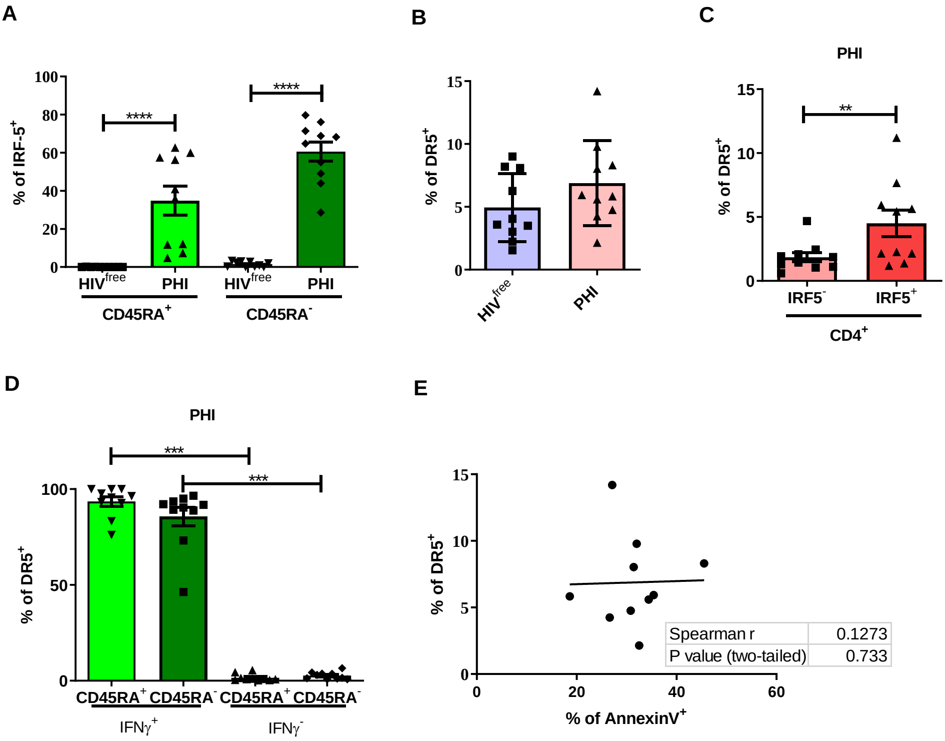

Supplemental Figure 2: DR5 expression in IRF-5⁺ cells. Graphs show a) the percentage of CD45RA^{+} and CD45RA^{-} CD4^{+} T cells expressing IRF-5 and b) the percentage of DR5^{+} CD4^{+} T cells in PHI and HIV^{free} individuals; c) the percentage of IRF5 expressing DR5^{+} CD4^{+} T cells, and d) the percentage of IRF5^{+} DR5^{+} CD4 T cells expressing CD45RA$^{+/-}$ IFNɣ$^{+/-}$ CD4^{+} T cells in PHI patients. Data are presented as the mean ± SD. The Mann-Whitney *U*-test and the Kruskal-Wallis test followed by the Dunn's multiple comparison test were used to determine statistical significance, * p <0.05, ** p< 0.01, *** p<0.001, n=10. e) Graph represents correlation between Annexin V and DR5 expressions in memory CD4^{+} T cells from PHI patients. The Spearman r test was used to determine statistical significance, * p <0.05, n=10.

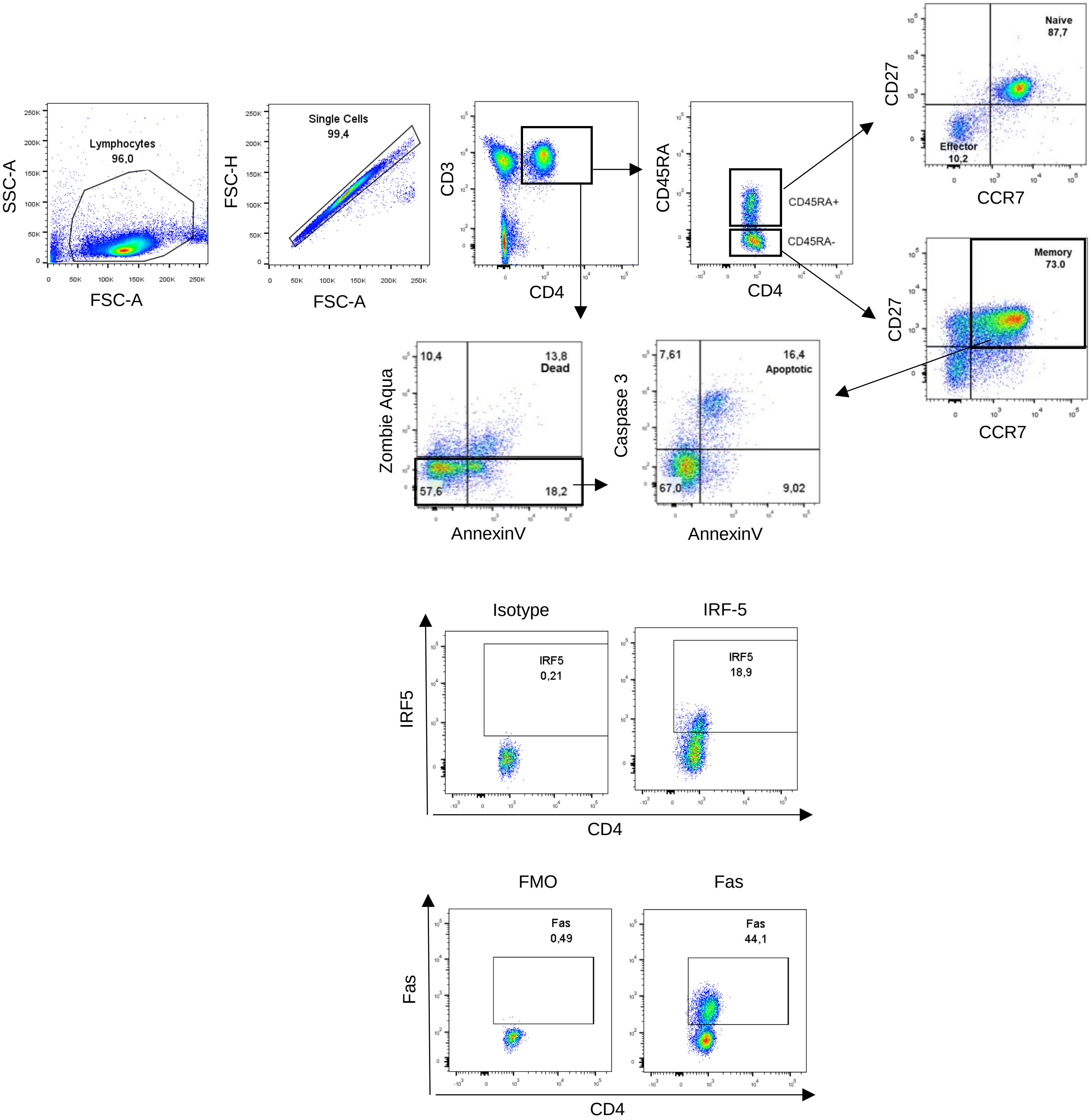

Supplemental Figure 3: Representative FACS plots showing the gating strategy used to determine the frequency of apoptotic, dead, IRF-5^{+}, and Fas^{+} CD4^{+} T cells or to gate naïve, effector and memory CD4^{+} T cells from ART HIV-1^{+}, EC, and HIV^{free} donors.

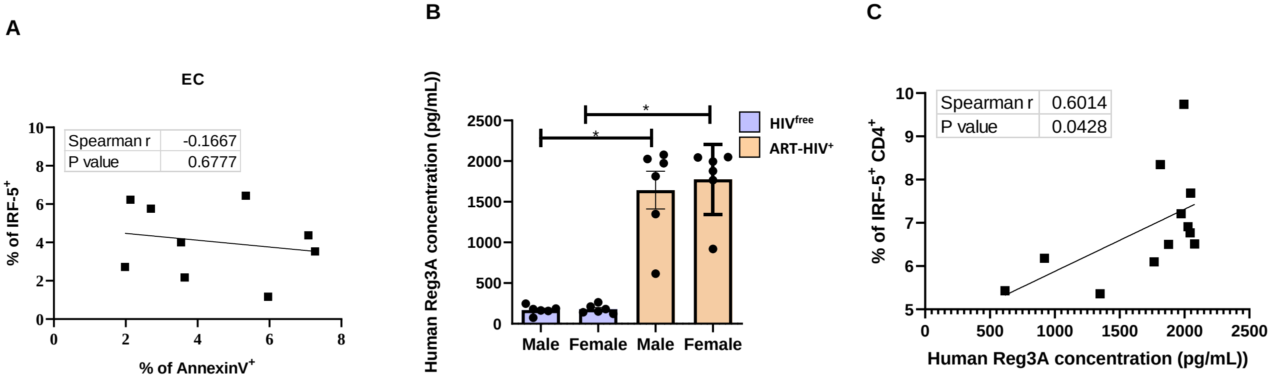

Supplemental Figure 4: IRF-5 expression does not correlate with Annexin V expression in EC but correlates with REG3α concentration in the serum of ART HIV-1⁺ donors. a) Graph represents correlation between Annexin V and IRF5 expressions in memory CD4⁺ T cells from EC. The Spearman r test was used to determine statistical significance, * p <0.05, n=10. b-c) The concentration of REG3α in the donors' serum was assessed by ELISA. Graphs show b) REG3α concentrations in the serum of male and female ART HIV-1⁺ participants, and c) the correlation between IRF-5 expression and REG3α serum concentrations. Data are presented as the mean ± SD. The Friedman's test and the Dunn's multiple comparison test were used to determine statistical significance, * p <0.05, n=6. For c), the Spearman r test was used to determine statistical significance, * p <0.05, n=12 (6 female and 6 male donors).

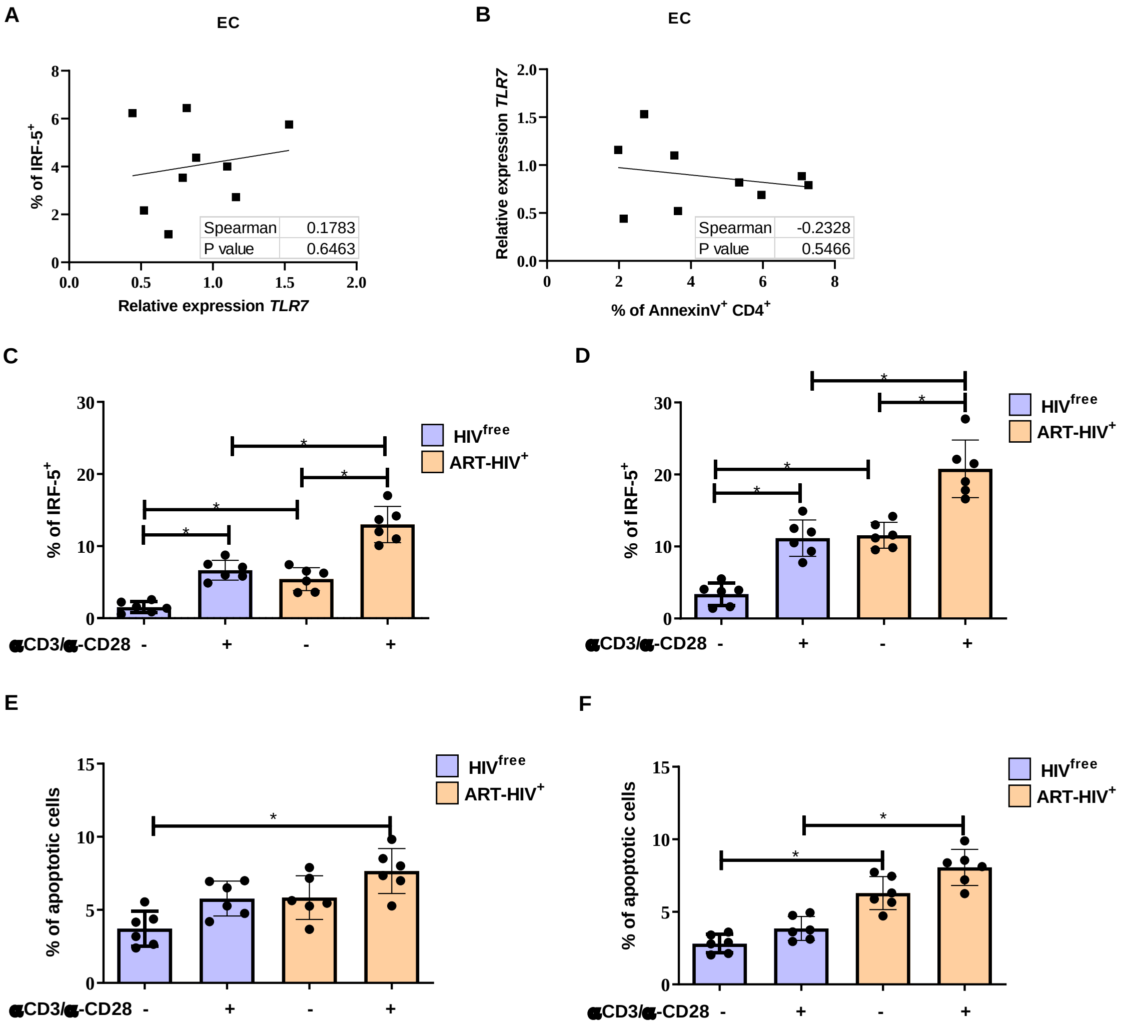

Supplemental Figure 5: Graphs represent correlations between a) *TLR7* and IRF-5 expressions and b) *TLR7* and Annexin V expressions in memory $CD4^+$ T cells from EC. Spearman r test was used to determine statistical differences * p <0.05, n=9. c-f) Purified $CD4^+$ T cells from HIV-$1^+$and HIV^{free} donors were stimulated for 24h with αCD3/αCD28. Graphs show c) the percentage of IRF-5^+ total $CD4^+$ T cells and the percentage of d) IRF-5^+, e) apoptotic cells, and f) dead cells in memory $CD4^+$ T cells. Data are presented as the mean ± SD. The Friedman's test and the Dunn's multiple comparison test were used to determine statistical significance, * p <0.05, n=6.

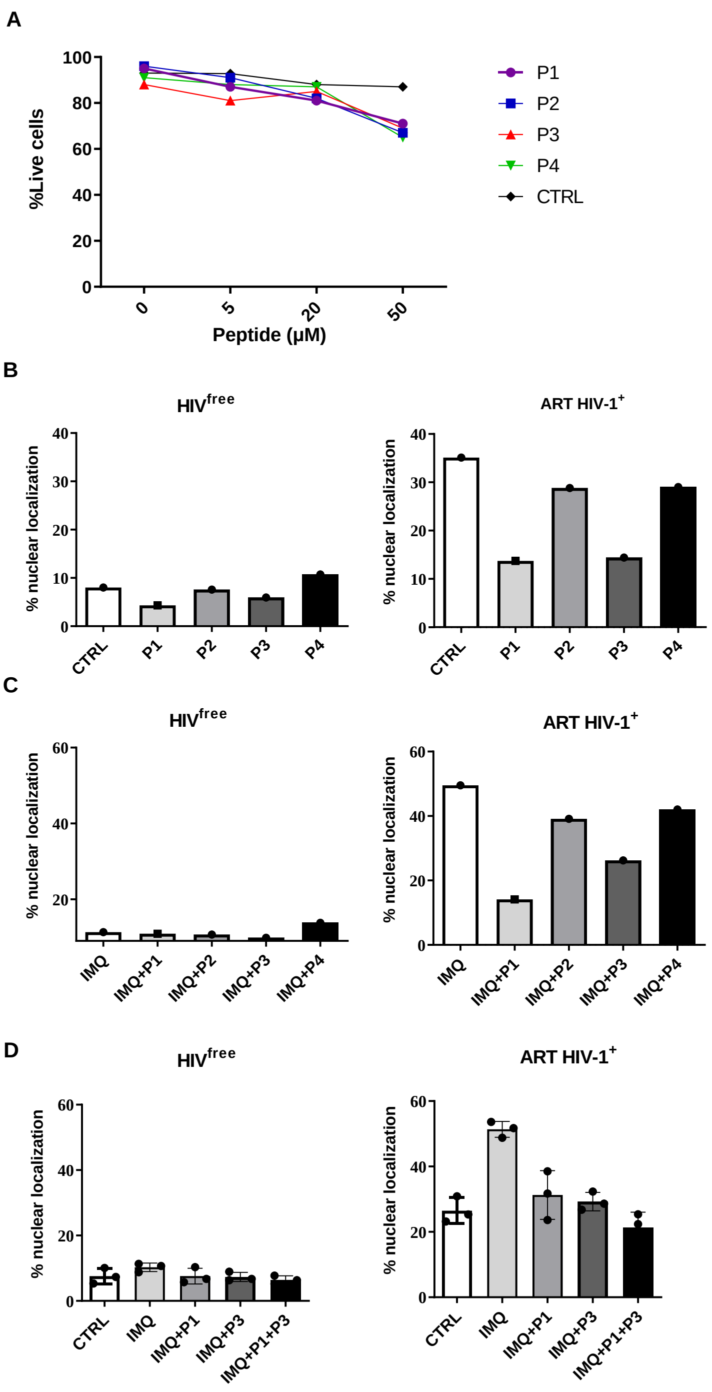

Supplemental Figure 6: IRF-5 inhibitory peptides limit Fas-mediated apoptosis in memory CD4⁺ T cells. Four different IRF-5 blocking peptides and the control peptide were tested for their cytotoxicity and their capacity to block IRF-5 in CD4⁺ T cells. Graphs show a) the percentage of live PBMCs from HIV^{free} donors upon incubation with IRF-5 inhibitory peptides at various concentrations, and b) IRF-5 nuclear localization in unstimulated and c,d) IRF-5 nuclear localization in IMQ-stimulated CD4⁺ T cells from ART HIV-1⁺ and HIV^{free} donors in the presence or absence of 10μM of the indicated peptides or peptide mix. Data are presented as the mean ± SD, n=3.

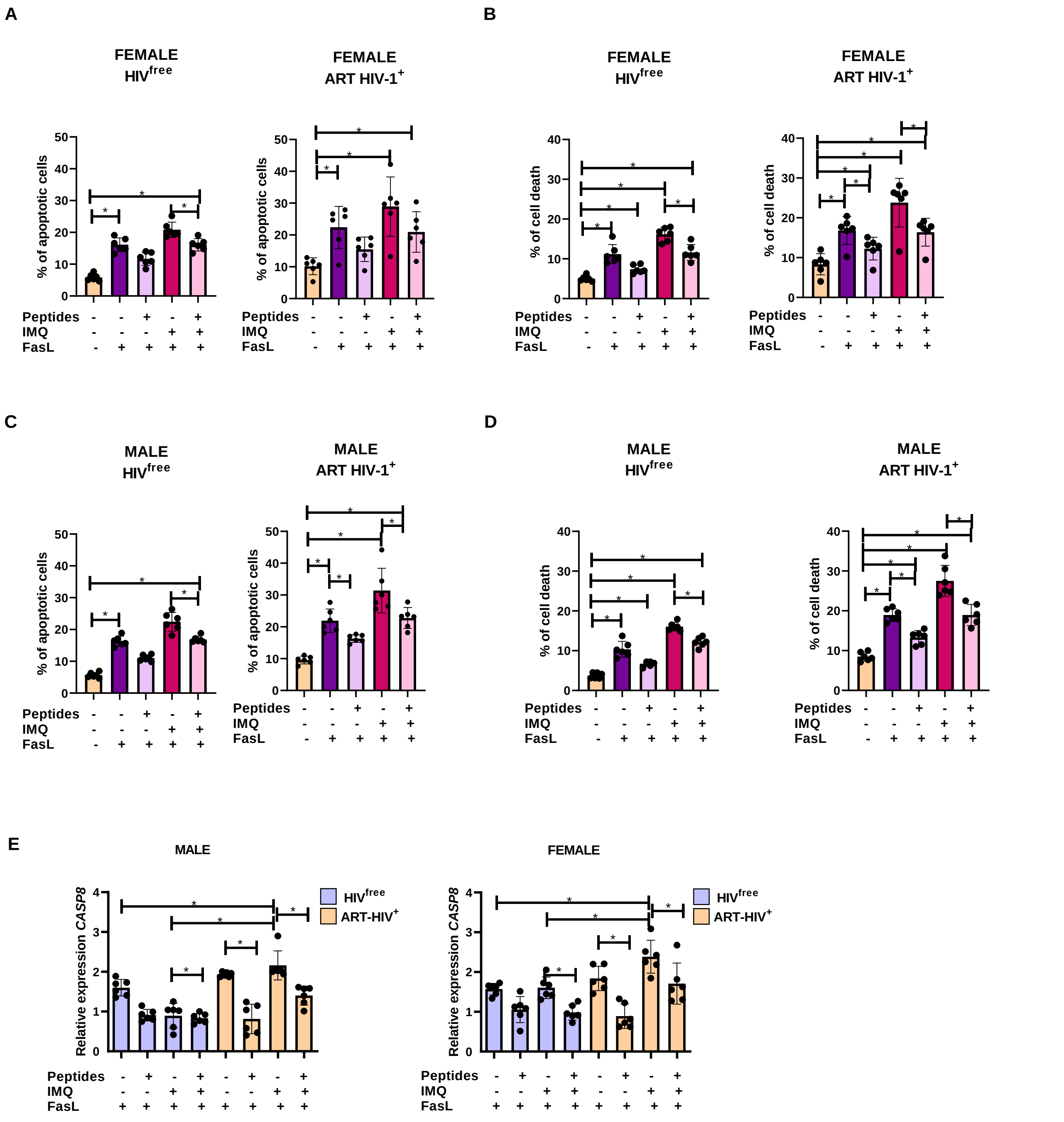

Supplemental Figure 7: CD4 T cells from male and female participants display similar responses. a) Purified $CD4^+$ T cells from female and male ART $HIV\text{-}1^+$ and HIV^{free} donors were treated with IMQ or medium alone; 12h later rFasL or medium were added to the culture and the cells were incubated for further 18h for a total of 30h incubation at 37°C. Graphs show the percentage of apoptotic memory $CD4^+$ T cells from a) female and c) male donors , the percentage of dead memory $CD4^+$ T cells from b) female and d) male participants, and e) *CASP8* mRNA levels in memory $CD4^+$ T cells from male (left graph) and female (right graph) donors after 30h stimulation. Data are presented as the mean ± SD. The Friedman's test and the Dunn's multiple comparison test were used to determine statistical significance, * $p < 0.05$, n=6.

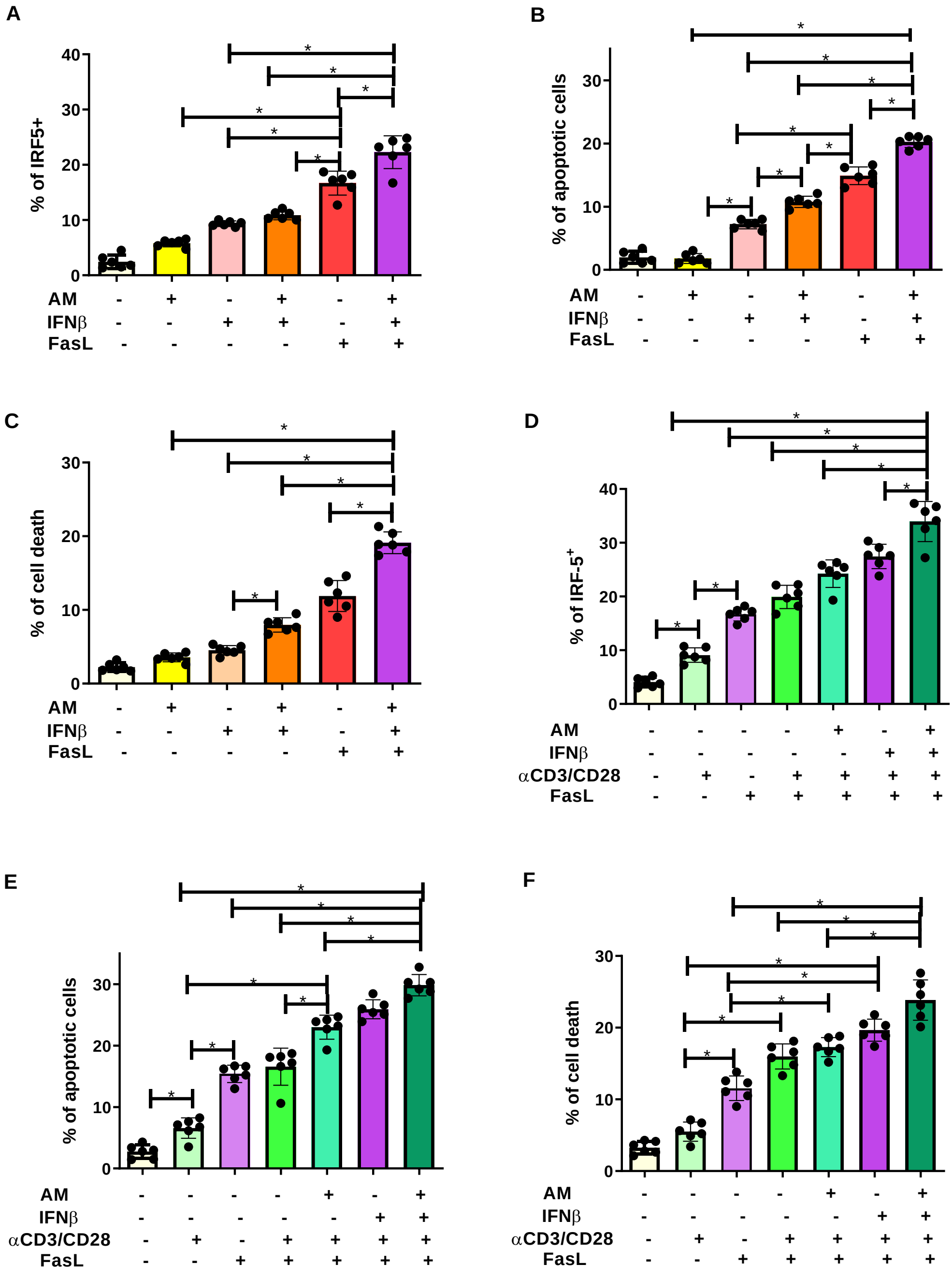

Supplemental Figure 8: Stimulation of memory $CD4^+$ T cells in the presence of DAMPs and IFNβ promotes the upregulation of TLR7 and predisposes cells to Fas-mediated apoptosis. Purified $CD4^+$ T cells from HIV^{free} individuals were treated *in vitro* for 24 h with 10 ng/ml IFNβ in the presence or absence of 10% v/v supernatant containing apoptotic material (AM, supernatant of staurosporine-treated cells), before stimulation with αCD3/αCD28 for 24h. Graphs show a) the percentage of IRF-5^+, b) the percentage of apoptotic and c) the percentage of death memory $CD4^+$ T cells. $CD4^+$ T cells from HIV^{free} individuals were treated as described above and additionally incubated with rFasL for further 18h after αCD3/αCD28 stimulation. Graphs show d) the percentage of IRF-5^+, e) the percentage of apoptotic and f) the percentage of death memory $CD4^+$ T cells. Data are presented as the mean ± SD. The Friedman's test and the Dunn's multiple comparison test were used to determine statistical significance, * $p < 0.05$, n=6.
